# Chloroplast genomes elucidate diversity, phylogeny, and taxonomy of *Pulsatilla* (Ranunculaceae)

**DOI:** 10.1038/s41598-020-76699-7

**Published:** 2020-11-13

**Authors:** Qiu-jie Li, Na Su, Ling Zhang, Ru-chang Tong, Xiao-hui Zhang, Jun-ru Wang, Zhao-yang Chang, Liang Zhao, Daniel Potter

**Affiliations:** 1 College of Life Sciences, Northwest A&F University, Yangling, 712100 China; 2 Herbarium of Northwest, A&F University, Yangling, 712100 China; 3 College of Life Sciences, Tarim University, Alaer, 843300 China; 4 College of Life Science, Shaanxi Normal University, Xi’an, 710062 China; 5 Department of Plant Sciences, MS2, University of California, Davis, CA 95616 USA

**Keywords:** Plant sciences, Systems biology

## Abstract

*Pulsatilla* (Ranunculaceae) consists of about 40 species, and many of them have horticultural and/or medicinal value. However, it is difficult to recognize and identify wild *Pulsatilla* species. Universal molecular markers have been used to identify these species, but insufficient phylogenetic signal was available. Here, we compared the complete chloroplast genomes of seven *Pulsatilla* species. The chloroplast genomes of *Pulsatilla* were very similar and their length ranges from 161,501 to 162,669 bp. Eight highly variable regions and potential sources of molecular markers such as simple sequence repeats, large repeat sequences, and single nucleotide polymorphisms were identified, which are valuable for studies of infra- and inter-specific genetic diversity. The SNP number differentiating any two *Pulsatilla* chloroplast genomes ranged from 112 to 1214, and provided sufficient data for species delimitation. Phylogenetic trees based on different data sets were consistent with one another, with the IR, SSC regions and the barcode combination *rbcL* + *matK* + *trnH-psbA* produced slightly different results. Phylogenetic relationships within *Pulsatilla* were certainly resolved using the complete cp genome sequences. Overall, this study provides plentiful chloroplast genomic resources, which will be helpful to identify members of this taxonomically challenging group in further investigation.

## Introduction

DNA barcoding is an effective tool to identify many plant species rapidly and accurately^1–4^. However, there is no single universal barcode that can be successfully used to identify all plants to the species level^5^. Consequently, two alternative strategies have been proposed to distinguish among plant species: the first one is the use of complete chloroplast genomes^6^, named ‘super-barcoding’, and the second one is an approach that involves searching for mutational hotspots^7^, or using comparative plastid analyses to find loci with suitable species-level divergence^8,9^. Analyses of entire chloroplast genome sequences provide an effective way to develop both of these strategies.

In most angiosperms, the chloroplast genomes are inherited maternally and have a consistent structure, including two inverted repeats (IR), one large (LSC) and one small (SSC) single copy region. The chloroplast genome always contains 110–130 genes that exhibit a range of levels of polymorphism^4,5^. Thus, chloroplast genome sequence data are extremely valuable for studies of plant population genetics, phylogeny reconstruction, species identification, and genome evolution^2,4–6^.

The Ranunculaceae is a large family, which includes approximately 59 genera and 2500 species. Many plants of Ranunculaceae are pharmaceutically important^10^. The genus *Pulsatilla* Adans. consists of about 40 species which are distributed in temperate subarctic and mountainous areas of the Northern Hemisphere^10^. There are always long, soft hairs covering plants of *Pulsatilla* species. Most of the flowers of *Pulsatilla* are large and showy, and therefore the genus has horticultural importance^11,12^. The flowers are solitary and bisexual. In one flower, there are always six tepals, numerous stamens and carpels, with the outermost stamens resembling degenerated petals, excluding *P. kostyczewii*^11,13–15^.

In China, there are eleven species of *Pulsatilla*. Some species of *Pulsatilla* have been used in traditional Chinese medicine for many years, such as for “detoxification” or “blood-cooling”, because *Pulsatilla* species contain numerous secondary metabolites, including phytosterols, triterpenoid saponins and anthocyanins^16^. At the same time, all members of *Pulsatilla* produced the lactone protoanemonin^17–19^.

In Europe, some species of *Pulsatilla* are rare, endangered and endemic. Those taxa are protected due to their small populations and disappearing localities, and those species have been placed on the Red Lists of Endangered Species^20^.

Taxonomically, *Pulsatilla* is an especially complex and challenging group. In all treatments published before, three subgenera have been recognized: subgenus *Kostyczewianae* (only one species), subgenus *Preonanthus*, and the largest subgenus *Pulsatilla*. However, the intragenic morphological variability of *Pulsatilla* was especially complicated^12^. The recognition and identification of wild *Pulsatilla* species is particularly difficult based on traditional approaches.

Molecular markers are significant to explore the phylogenetic relationships of the genus *Pulsatilla*. Phylogenetic relationships between *Pulsatilla* and closely related genera have been dedicated during the past years^21–25^. Previous studies have attempted to identify these species among *Pulsatilla* with universal molecular markers, but the species resolution was relatively low^15^.

In this study, we present seven complete cp genomes from two subgenera of *Pulsatilla* obtained through next-generation sequencing (NGS) and genomic comparative analyses with four previously published cp genome sequences of *Pulsatilla* from NCBI, with *Anemoclema glaucifolium* as the outgroup. We identify microsatellites (SSRs), larger repeat sequences, and highly variable regions, with the aim of developing DNA barcodes and testing the feasibility of phylogenetic analyses of *Pulsatilla* using the chloroplast genome.

## Results and discussion

### Chloroplast genome features

**Figure 1:**
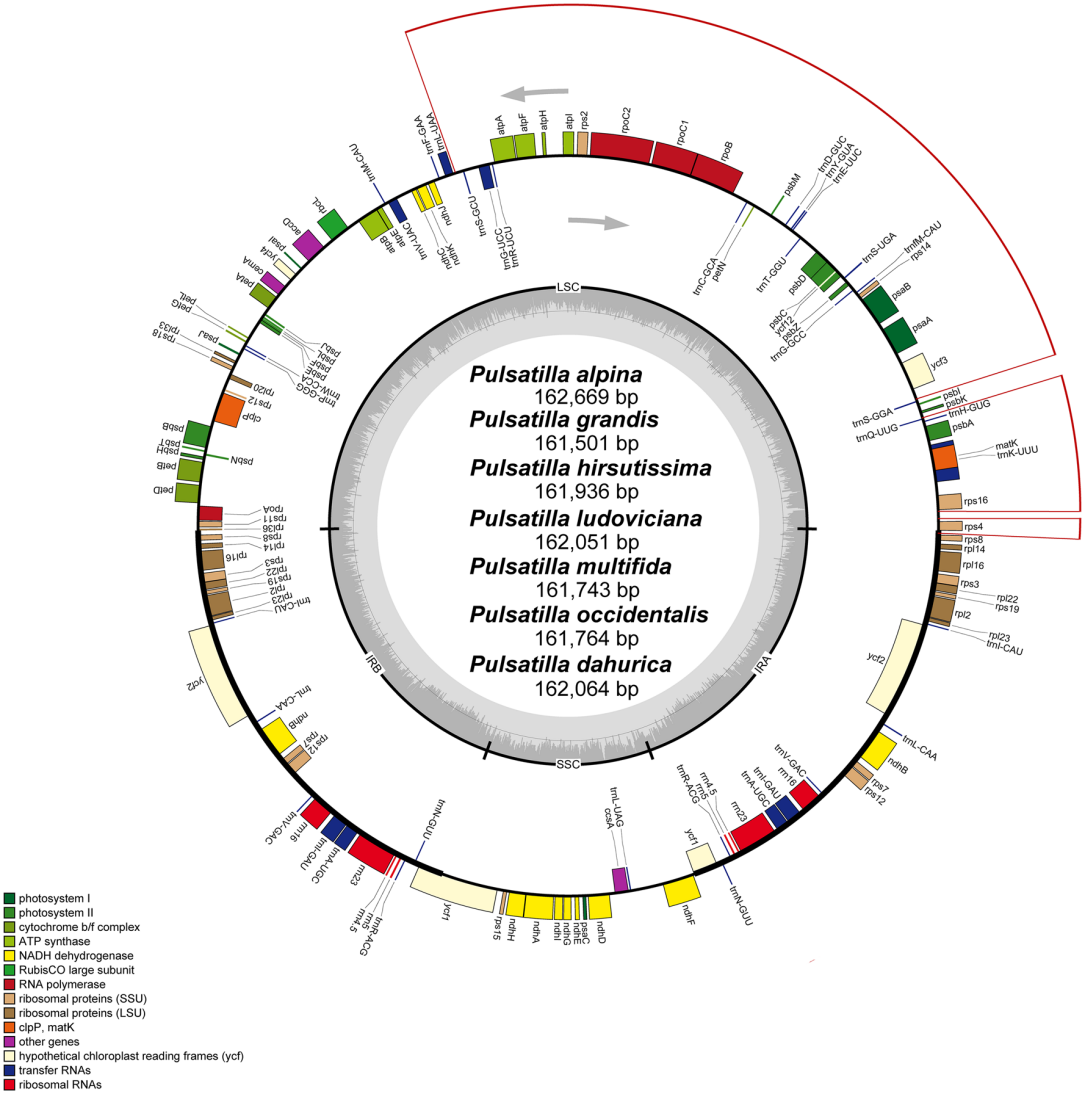
Gene map of the *Pulsatilla* chloroplast genome. Dashed area in the inner circle indicates the GC content of the chloroplast genome. LSC, SSC and IR mean large single copy, small single copy and inverted repeat, respectively. Genes belonging to different functional groups are color-coded as indicated by icons on the lower left corner. The red line on the outside of the gene is three inversions.

We have obtained 1.95 Gb of average NGS clean data for each species, with minimum and maximum values of 1.14 Gb (*P. dahurica*), and 3.56 Gb (*Pulsatilla alpina*), respectively. The read number for each species ranged from 6,468,944 (*P. dahurica*) to 15,816,765 (*P. alpina*). The average length of the reads was 150 bp on the Illumina Sequencing System. The seven new *Pulsatilla* cp genomes ranged from 161,501 bp (*P. grandis*) to 162,669 bp (*P. alpina*) in length and 151.5 × to 503.4 × coverage. These seven novel *Pulsatilla* cp genome sequences were submitted to GenBank (Tables 1, 2). Their quadripartite structure is similar to the majority of cp genomes of land plants, which are composed of a pair of IRs (31,184–31,416 bp), separated by the LSC (81,615–82,149 bp) and SSC (of 17,431–17,908 bp) regions^26,27^ (Fig. 1; Table 2). Previous studies of other angiosperm groups have found that chloroplast genomes are conserved^28^ or highly polymorphic^29,30^. These genomes which we reported are highly conserved in gene order, gene content and intron number, which is in accordance with the results from many other taxa^26,27^. However, in some taxa, e.g. *Amorphophallus* of Araceae, some genes were lost^31^, and in others, e.g. *Pelargonium*^32^, the structure and gene order diverges from what is reported here and in most other angiosperms. The cp genomes of *P. alpina*, *P. grandis*, *P. hirsutissima*, *P. ludoviciana*, *P. multifida* and *P. occidentalis* had the same GC content of 37.6%, while *P. dahurica* had a subtle difference (37.5%) compared with the others.

**Table 1:** Voucher information and GenBank accession numbers for *Pulsatilla* and outgroups.

Taxon	Location	Date	Herbarium	Accession	SRA accession
P. grandis*	Europe MoaiJuni	1904	US	MN025344	SRR12822481
P. multifida*	U.S.S.R.	1957	US	MN025347	SRR12822474
P. alpina*	America Graubunden	1936	US	MN025343	SRR12822486
P. occidentalis*	America Siskiyon Calfornia	1943	US	MN025348	SRR12822484
P. ludoviciana*	America Albany county	1898	US	MN025346	SRR12822477
P. hirsutissima*	America	–	US	MN025345	SRR12822480
P. dahurica*	China	2014	WUK	MN025349	SRR12822482
*P. patens*	NA	NA	NA	KR_297058	NA
*P. pratensis*	NA	NA	NA	KR_297060	NA
*P. vernalis*	NA	NA	NA	KR_297062	NA
*P. chinensis*	NA	NA	NA	MG_001341	NA
*Anemoclema glaucifolium*	NA	NA	NA	MH_205609	NA

Species with asterisks were collected by this study, whereas others were obtained from Genbank.

*NA* not applicable.

**Table 2:** Summary of complete chloroplast genomes of *Pulsatilla.*

Species	Number of reads	Average depth of coverage (×)	Size (bp)	Length (bp)			Coding (bp)	Non-coding (bp)	GC%
				LSC	SSC	IR			
*P. grandis*	13,012,108	194.8	161,501	81,672	17,431	31,199	78,377	78,377	37.6
*P. multifida*	11,405,519	151.5	161,743	81,653	17,648	31,221	81,800	81,800	37.6
*P. alpina*	15,816,765	367.1	162,669	82,149	17,688	31,416	81,246	81,246	37.6
*P. occidentalis*	12,554,200	276.9	161,764	81,615	17,755	31,197	79,089	79,089	37.6
*P. ludoviciana*	9,251,830	257.5	162,051	81,860	17,771	31,210	79,280	79,280	37.6
*P. hirsutissima*	13,337,339	503.4	161,936	81,866	17,702	31,184	80,206	80,206	37.6
*P. dahurica*	6,468,944	410.1	162,064	81,688	17,908	31,234	79,114	79,114	37.5

### Chloroplast genome comparison

In most angiosperms, the IR regions of cp genomes of angiosperms are highly conserved, but the expansion and contraction of IR region boundaries are ever present^33,34^. At the same time, several lineages of land plant chloroplast genomes show great structural rearrangement, even loss of IR regions or some gene families^35^. The expansion and contraction in IRs are significant evolutionary events, because they can change gene content and chloroplast genome size^30,36^. Expansion of the IRs has been reported in Araceae^36,37^. Sometimes, the size of LSC increases and that of SSC decreases, becoming only 7000 bp in *Pothos*^38^. At the same time, a linear chloroplast genome was also reported in some groups, e.g. maize^35,39^. Expansion and contraction of the IR regions can also lead to duplication of certain genes or conversion of duplicate genes to single copy, respectively^30,36^. Changes in the size of the IRs can also cause rearrangement of the genes in the SSC as recently observed in *Zantedeschia*^36^.

**Figure 2:**
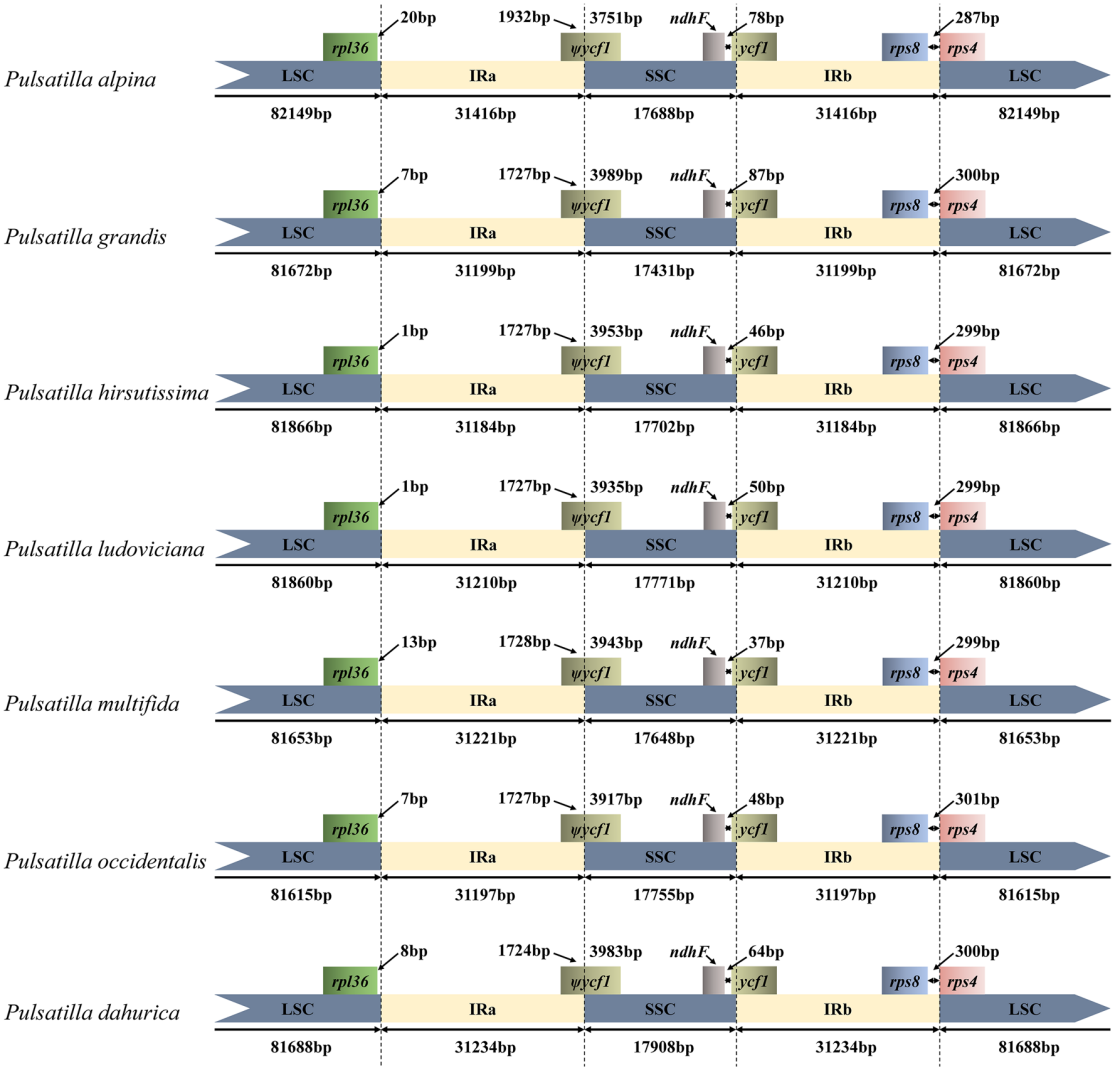
Comparisons of LSC, SSC, and IR region borders among the seven *Pulsatilla* chloroplast genomes.

The *Pulsatilla* chloroplast genomes were compared to previously published data and showed typical Anemoneae (Ranunculaceae) genome structure^37,39^. As reported for *Anemoclema*, *Anemone*, *Clematis* and *Hepatica*, the IR regions of genus *Pulsatilla* are roughly 4.4 kb longer than those of other genera of the family Ranunculaceae, such as *Aconitum*, *Coptis*, *Thalictrum*, *Megaleranthis*, *Ranunculus*, and *Trollius*^37,39^. The gene orders located within the IR-SSC and IR-LSC boundaries are similar among tribe Anemoneae but different from those of other genera of Ranunculaceae (Fig. 2, Fig. S1).

We compared the IR/SC boundary regions of *Pulsatilla*, and the junction positions are very similar and conserved within genus *Pulsatilla*. In the four boundary regions (LSC, IRa, SSC, IRb) of seven *Pulsatilla* cp genomes, the LSC/IRa and IRb/LSC border was in the intergenic region, and the adjacent genes is *rps36*, *rps8* and *rps4*, respectively. The genes *ycf1* and*ψycf1* have crossed the SSC/IRb and IRa/SSC boundary, respectively, which was also found in Monsteroideae (Araceae)^28^. The pseudogene *ycf1* has been found in other groups^30,36^. The IR regions were highly conserved, with nucleotide diversity values in those regions less than 2%.

### Sequence divergence

**Figure 3:**
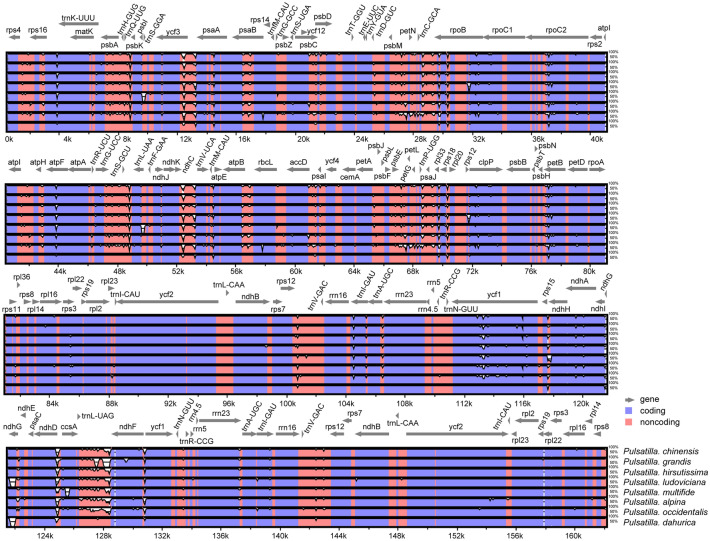
Visualized alignment of the eight *Pulsatilla* chloroplast genomes. The mVISTA-based identity plots show the sequence identity among the seven chloroplast genomes, with *P. chinensis* serving as a reference. Blue represents coding regions, and pink represents non-coding regions.

Multiple alignments of plastid genomes were performed to investigate levels of genome divergence. Based on MAFFT analysis, there are three inversions in LSC of *Pulsatilla*, same as the tribe Anemoneae (Fig. 1, Fig. S1)^37^. The mVISTA analysis has revealed high sequence similarity across the coding region and there exists more variability in non-coding regions. Sequence identity among the seven species was 96.68–98.66%. The number of nucleotide substitutions and sequence distance (Pi) were the highest (1214, 0.0063) between *P. alpine* and *P. dahurica*, with the lowest (112, 0.0005) between *P. vernalis* and *P. patens* (Fig. 3; Table 3).

**Table 3:** Numbers of nucleotide substitutions and sequence distance (Pi) in eleven complete cp genomes.

	*P. chinensis*	*P. dahurica*	*P. patens*	*P. vernalis*	*P. hirsutissima*	*P. ludoviciana*	*P. pratensis*	*P. grandis*	*P. multifide*	*P. alpina*	*P. occidentalis*
*P. chinensis*		0.0028	0.0027	0.0029	0.0030	0.0035	0.0024	0.0034	0.0028	0.0058	0.0064
*P. dahurica*	563		0.0033	0.0034	0.0034	0.0038	0.0029	0.0039	0.0033	0.0063	0.0068
*P. patens*	558	642		0.0005	0.0011	0.0015	0.0019	0.0028	0.0010	0.0046	0.0052
*P. vernalis*	572	638	112		0.0011	0.0015	0.0019	0.0028	0.0010	0.0046	0.0051
*P. hirsutissima*	652	676	205	229		0.0009	0.0020	0.0029	0.0008	0.0048	0.0053
*P. ludoviciana*	685	735	290	303	200		0.0024	0.0034	0.0013	0.0052	0.0058
*P. pratensis*	470	516	378	383	392	547		0.0020	0.0019	0.0050	0.0055
*P. grandis*	662	750	586	577	604	701	414		0.0029	0.0059	0.0064
*P. multifide*	650	735	215	233	198	284	463	527		0.0046	0.0052
*P. alpina*	1212	1214	915	903	916	1007	964	1128	908		0.0045
*P. occidentalis*	1207	1293	979	968	1006	1081	1046	1219	930	869	

The uppertriangle shows the number of nucleotide substitutions and the lower triangle indicates the number of sequence distance in complete cp genomes.

### Identification of highly variable regions

**Figure 4:**
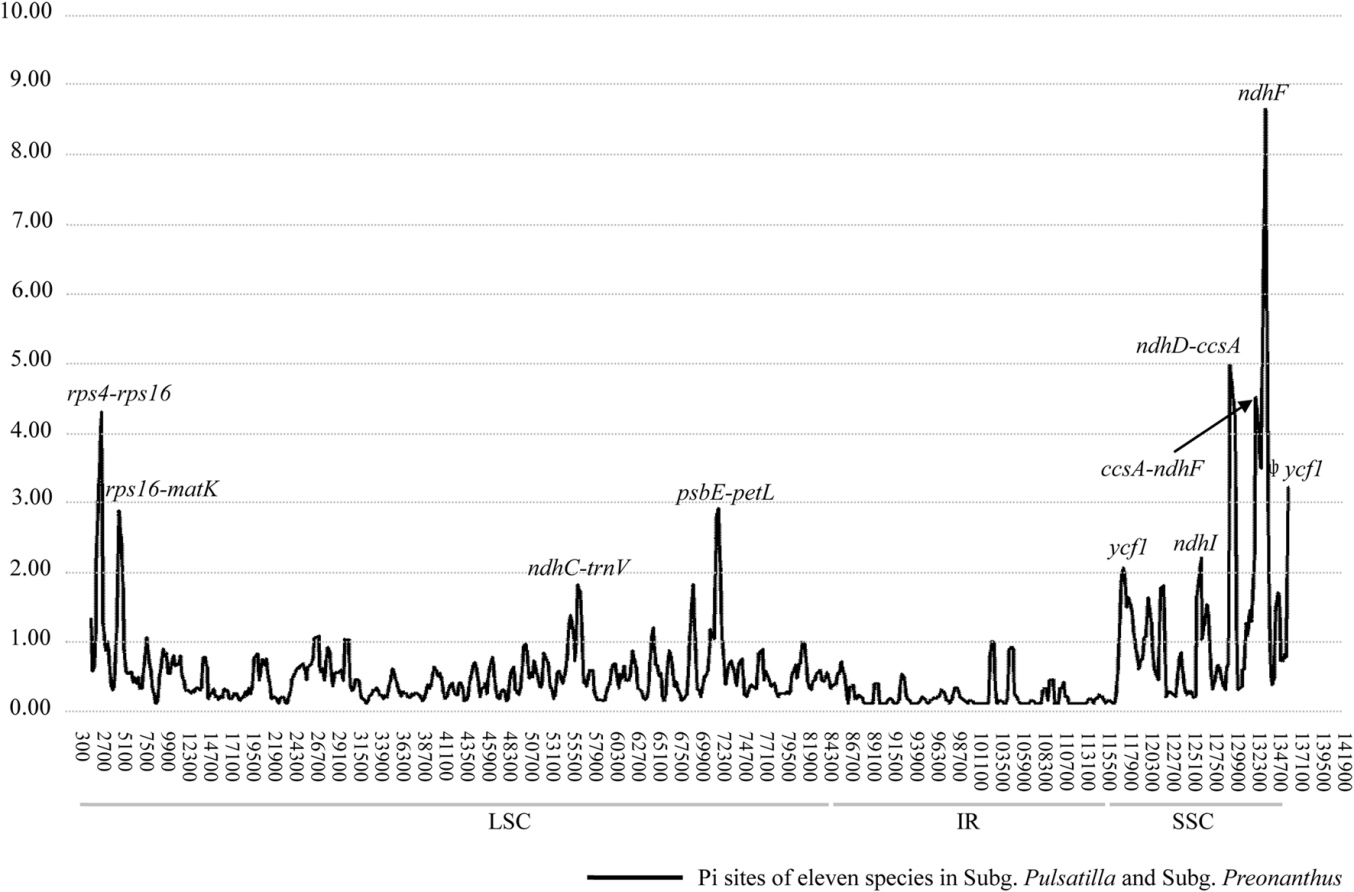
Sliding window analysis of the whole chloroplast genomes of *Pulsatilla* taxa.

Chloroplast genome markers, especially several universal chloroplast regions, have been widely used in plant systematics and identification at multiple taxonomic levels. Highly suitable polymorphic chloroplast loci have been identified and designed as unique markers in different groups^28,40^. However, relationships within the genus *Pulsatilla* have not been well resolved because of the low polymorphism of these universal markers^15^. In order to facilitate identification of closely related species of *Pulsatilla*, we sought to identify highly variable regions of the chloroplast genome, as previously described^9,27,41–44^. As a result, we identified nine divergent hotspot regions, including six intergenic spacer regions (*rps4-rps16*, *rps16-matK*, *ndhC-trnV*, *psbE-petL*, *ndhD-ccsA*, *ccsA-ndhF*) and four protein-coding regions (*ycf1*, *ndhF*, *ndhI*) (Fig. 4; Table 4). Most commonly employed loci, e.g. *trnL-trnF*, *trnH-psbA* were not selected in our finding. The nine highly variable regions included 684 variable sites, including 181 indels. However, these indels are not suitable for the phylogenetic inference because Maximum likelihood model used only substitutions not indels^28^. Their nucleotide diversity values ranged from 0.00802 to 0.02212. The region of *ccsA-ndhF* showed the highest variability, the next most variable regions were *rps4-rps16*, *ndhC-trnV*, and *psbE-petL*. The diversity level of two protein-coding regions (*ycf1*, *ndhF*) was the lowest.

**Table 4:** Sequence characteristics of eight high variable regionsamong eleven complete cp genomes of *Pulsatilla*.

Region	Aligned length	Variable sites		Indels		Nucleotide diversity (Pi)
		No	%	No	Length range	
*rps4-rps16*	986	22	2.23	11	1–44	0.01497
*rps16-matK*	1985	62	3.12	23	1–79	0.00998
*ndhC-trnV*	1484	61	4.11	18	1–34	0.01502
*psbE-petL*	1469	70	4.77	24	1–139	0.01140
*ndhD-ccsA*	732	39	5.33	10	1–87	0.04368
*ccsA-ndhF*	2757	196	7.11	69	1–43	0.02212
*ycf1*	5807	148	2.55	18	1–24	0.00802
*ndhF*	2345	86	3.67	8	1–18	0.00813

Among the nine divergent hotspot regions, the *ndhI* is difficult to align. There are large numbers of indels in *ndhI* and the intergenic spacer between *ndhI* and *ndhG*, these regions were not considered suitable for the phylogenetic inference of the *Pulsatilla*. Thus, we selected eight regions, four (*rps4-rps16*, *rps16-matK*, *ndhC-trnV*, *psbE-petL*) in the LSC and four (*ndhD-ccsA*, *ccsA-ndhF, ycf1*, *ndhF*) in the SSC, with relatively high variability as potential molecular markers for the study of species identification and phylogeny in *Pulsatilla*. Five hotpots were found in chloroplast genome of Veroniceae (Plantaginaceae), and two universal marker, *trnH-psbA* and *matK* were identified, respectively^45,46^. Ten highly variable regions were selected as potential molecular markers for *Fritillaria*, including *ycf1*^44,47^, which was also selected in this study. Sequences of these variable regions founded in this study could be regarded as potential molecular markers for species identification and evolutionary studies and have been shown to be valuable for studies in other groups (e.g., *Fritillaria*)^44^.

### SSRs and large repeat sequences

**Figure 5:**
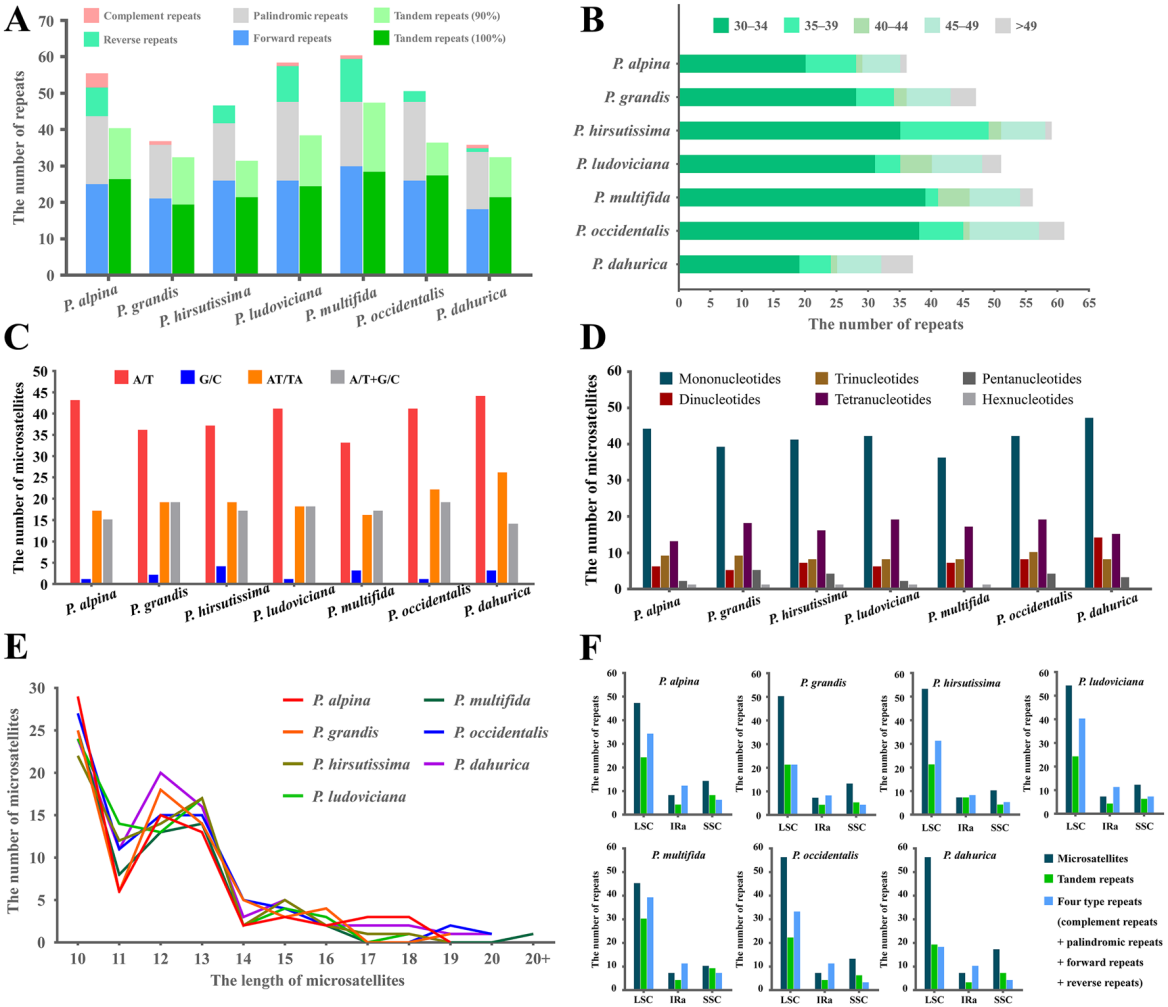
Analyses of repeated sequences in seven newly sequenced chloroplast genomes. (**A**) Number of five repeat types; (**B**) frequency of four repeats by length; (**C**) frequency of microsatellites by base composition; (**D**) frequency of microsatellites by types; (**E**) frequency of microsatellites by length; (**F**) number of all repeats by location.

Oligonucleotide repeats play an important role for generating indels, inversion and substitutions^29^. Repeat sequences in the chloroplast genome could provide valuable information for understanding not only the sequence divergence but the evolutionary history of the plants^48–50^. We have detected five types of large repeats (forward, reverse, palindromic, complement and tandem repeats) in the seven *Pulsatilla* cp genomes. Among them, the most common repeat types are forward and palindromic repeats, followed by reverse repeats, and only little complement repeats were found in *Pulsatilla* cp genomes (Fig. 5A). Most of the repeats were short, ranging from 30–49 bp (Fig. 5B).

We also identified multiple microsatellite repeats, also known as simple sequence repeats (SSR) or short tandem repeats (STR)^49^. Due to their codominant inheritance and high variability, SSRs are robust and effective markers for species identification and population genetic analyses^49–53^. Most of the mononucleotide repeats were composed of A/T. The other microsatellites types were also dominated by AT/TA, with very little G/C (Fig. 5C). In this study, plentiful microsatellite loci were found through the comparative analysis of *Pulsatilla* cp genome sequences. In total, we detected six types of microsatellite (mononucleotide, dinucleotide, trinucleotide, tetranucleotide, pentanucleotide and hexanucleotide repeats) based on the comparison of seven *Pulsatilla* cp genomes (Fig. 5D). Each *Pulsatilla* cp genome had 69–87 microsatellites. The lengths of repeat motifs of these microsatellites ranged from 10 to 21 bp (Fig. 5E). Among the four structural regions in the cp genomes, most of the repeats and microsatellites were distributed in LSC, followed by SSC, and fewest in IRa/IRb (Fig. 5F), which were also reported in other studies in angiosperms^29,30^. These SSRs and repeat sequences are uncorrelated with genome size and phylogenetic position of the species^36^, but will provide important information for further studies of phylogenetic reconstruction and infra- and inter-specific genetic diversity^54,55^.

### Phylogenetic analyses

**Figure 6:**
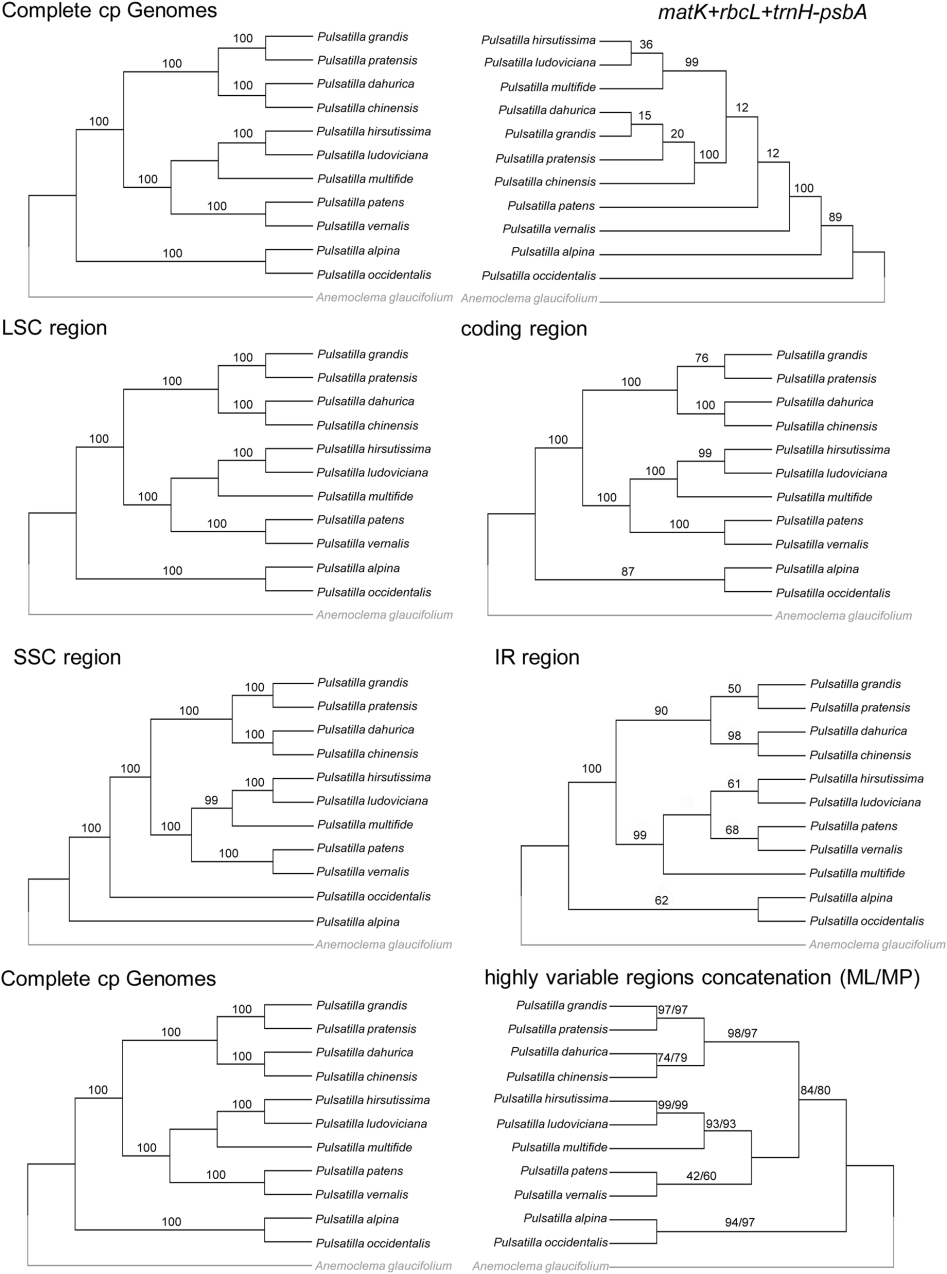
Phylogenetic relationships of the eleven *Pulsatilla* species inferred from maximum likelihood (ML). Including whole chloroplast genome, *rbcL* + *matK* + *trnH-psbA*, LSC region, coding region, SSC region, IR region, and the concatenation of the eight highly variable regions mentioned in Table 4 (Numbers above nodes are support values with ML bootstrap values on the left, and MP bootstrap values on the right).

Chloroplast genomes have been widely used and have made significant contributions to phylogeny reconstruction at different taxonomic levels in plants^7,9,24,35^. To better clarify the evolutionary relationships within *Pulsatilla*, we used each data set to construct phylogenetic trees using the ML analytical methods. We also construct phylogenetic trees with those eight highly variable regions using the ML, MP analytical methods. All tree topology structures were identical. Therefore, here we presented the phylogenetic studies using the ML tree with the support values from the MP analyses recorded at the corresponding nodes (Fig. 6). The phylogenetic tree based on all data sets (except the IR and SSC regions) from the complete plastid genome sequences yielded the same topology. The phylogenetic tree based on chloroplast genome differed from that of the DNA barcode combination *rbcL* + *matK* + *trnH-psbA*, but with higher support values. The phylogenetic trees based on data from complete plastid genome sequences showed that the species of *Pulsatilla* formed a monophyletic group which in turn includes two strongly supported (bootstrap = 100) clades. One clade comprised *P. alpina* and *P. occidentalis*, members of subg. *Preonanthus*. The other comprised two subclades: (1) members of *P. hirsutissima*, *P. ludoviciana*, *P. multifidi*, *P. patens* and *P. vernalis*, and (2) species of *P. chinensis*, *P. dahurica*, *P. grandis* and *P. pratensis*. All the species of the two subclades are members of the subg. *Pulsatilla.* These results were congruent with our former results based on universal markers^15^.

In phylogenetic analyses, compared to the combination of barcodes, the full chloroplast genome sequence data formed distinct clades with high bootstrap support, improving the inadequate resolution of barcodes combination. The LSC regions and coding regions have the same topology structures with robust support. However, sequencing of genomic DNA is still expensive. It is necessary to utilize variation within chloroplast regions for rapid species-specific assay^5,9,31,33,42^. Here we found that phylogenetic inference based on highly variable regions yielded a tree with the same topology as the one recovered based on complete chloroplast genome sequences, demonstrating the high utility of hotspots of variability for species identification and phylogenetic analysis. More samples and laboratory works are needed in the future to increase the number of these variable regions available for study.

## Conclusions

In this study, we generated complete chloroplast genomes of seven species of *Pulsatilla* and compared them to four previously published cp genome sequences of *Pulsatilla*. Chloroplast genomes of *Pulsatilla* share many features with those of other angiosperms. Informative differences between cp genomes of *Pulsatilla* were most evident in inversions in the large single copy region and expansion of the inverted repeat region. We identified multiple potentially valuable genetic markers, including large repeat sequences, numerous SSRs, and eight highly variable regions. Genetic markers have provided a reference for the improvement of plants fingerprints and the identification of similar *Pulsatilla*. In addition, it will be better to construct the phylogenetic relationships of *Pulsatilla* species using these highly variable regions and genetic markers in the future. Totally, this study provides a basis for future studies of horticultural cultivation, conservation, population genetics, phyletic evolution, development of DNA barcodes, and diverse research in *Pulsatilla.*

## Materials and methods

### Plant material, DNA extraction and sequencing

Fresh leaves of *P. dahurica* were collected from Jilin province of China and dried with silica gel. Dry leaves of other six *Pulsatilla* species were taken from herbarium specimens. We extracted total genomic DNA with the DNeasy Plant mini kits (QIAGEN, Guangzhou, China). The genomic DNA was sequenced using the Illumina Miseq platform (Illumina, San Diego, CA, USA).

### Chloroplast genome assembly and annotation

Whole chloroplast genome sequencing was done for the seven species of *Pulsatilla*. For each species, high-quality Illumina sequencing reads were assembled into scaffolds with de novo sequence assembly software Spades, SOAPdenovo and CLC Genomics Workbench v.6.5 (CLC Bio), respectively^56^. We checked the contigs against the reference genome of *P. chinensis* (MG001341), using BLAST (https://blast.ncbi.nlm.nih.gov/) and oriented aligned contigs according to the reference genome. We mapped all the raw reads back to assembled sequences to check the assembly and then constructed the complete cp genomes using Geneious v.9.0^57^. We submitted all the newly sequence data in raw format (fastq) and obtained SRA accesions (Table 1).

Annotations of cp genome sequences were performed using Plastid Genome Annotator (https://github.com/quxiaojian/PGA)^58^ and adjusted in Geneious v.9.0. To verify the accuracy of the annotations, we also used GeSeq^59^ to annotate each chloroplast genome in this study. We checked every boundary of tRNAs using tRNAscan-SE v.2.0^60^. The circular genome maps were generated in OGDRAW (https://ogdraw.mpimp-golm.mpg.de/)^61^.

### Genome comparisons

We aligned the cp genomes of *Pulsatilla* using multiple alignment of MAFFT v7^62^ and manually edited in Geneious v.9.0. The contraction and expansion of inverted repeat regions were also examined among the seven species (excluded *P. chinensis*, MG001341) of the genus *Pulsatilla* using Irscope^63^. Then, we performed multiple alignments of the eight genomes of *Pulsatilla* in the mVISTA program^64^ under Shuffle-LAGAN mode, with default parameters for other options, using the annotation genome of *P. chinensis* as a reference, with the aim of comparing and visualized the similarities and differences among different plastid genomes.

To analyse chloroplast genome organisation and gene arrangement, we perform the analyses of collinear blocks with Mauve v 2.3.1^65^ plugin in Geneious v.9.0, including only one copy of the IR, assuming collinear genomes for the full alignment. Detailed gene inversions were identified by comparing the gene order of *Pulsatilla* samples and *Anemoclema* to *Berberis*.

To observe the plastid genome divergence and determine parsimony informative sites, we conducted sliding window analysis after alignment to determine the nucleotide diversity (Pi) of the cp genome using DnaSP v5, with 200 bp of step size and 600 bp window length^66^. We defined hotspots as those regions with a higher value of Pi. We computed the variable sites across the complete cp genomes and the sequence characteristics of hotspots by DnaSP v5.0.

### Repeated sequences identification

We identified repeat sequences, including palindromic, reverse and forward repeats, using the online software REPuter (https://bibiserv.cebitec.uni-bielefeld.de/reputer), with the following settings: Hamming distance of 3 and minimum repeat size of 30 bp^67^. We used the online program Tandem Repeats Finder (https://tandem.bu.edu/trf/trf.html) to find the tandem repeat sequences, in which the similarity percentage of two repeat copies was at least 90% and the minimal repeat size was 10 bp. The alignment parameters for match, mismatch, and indels were set at 2, 7, and 7, respectively. We identified microsatellites (SSRs) by MISA^68^ with thresholds of 10, 5, 4, 3, 3, and 3 for mono-, di-, tri-, tetra-, penta-, and hexa-nucleotide, respectively.

### Phylogenetic analyses

For the purpose of reconstructing the phylogenetic relationships, four published complete cp genome sequences from the genus *Pulsatilla* and *Anemoclema* were also included in our analyses. The monotypic genus *Anemoclema* (MH205609) was selected as the outgroup. Because molecular evolutionary rates among the different cp genome regions are diverse, analyses of phylogenetic relationships were performed based on the following seven datasets: (a) the complete cp genome sequences; (b) coding genes (CDS); (c) one inverted repeat (IR) region (IRb); (d) the large single copy region (LSC); (e) the small single copy region (SSC), (f) the consensus sequences of eight highly variable regions; and (g) the DNA barcodes combination (*rbcL* + *matK* + *trnH-psbA*). We applied Maximum Likelihood (ML) analysis for each of the seven datasets to construct tree-sets. Maximum Parsimony (MP) analyses were also applied for the consensus sequences of eight highly variable regions and the DNA barcodes combination.

We conducted ML analyses with RAxMLHPC2 v.8.0.9^69^ on the Cyberinfrastructure for Phylogenetic Research (CIPRES) Science Gateway v.3.3^70^. Then the analysis of 1000 rapid bootstrap replicates (-x) was followed by a search for the best-scoring ML tree in one program (-f a). The best-fit model for nucleotide and amino acid sequences were evaluated using jModelTest 2^71^. We applied the GTR + G model to nucleotide data for both bootstrapping and best-tree searching phases, with other parameters as the default settings. We performed the maximum Parsimony (MP) analysis on PAUP* v.4.0b10^72^. All the characters were treated as unordered and equally weighted. The heuristic search specified 1000 random sequence addition replicates with TBR branch swapping, saving only 10 trees per replicate. We obtained the strict consensus tree from all the most-parsimonious trees (MPTs) detected during the search. We calculated bootstrap percentages (BP) from 10,000 rapid bootstrap replicates, each comprising 10 random sequence addition replicates and saving only one tree per replicate.

## Supplementary information

Supplementary Figure S1.

Supplementary Table S1.

Supplementary Caption.

## References

[1] Hebert PDN, Cywinska A, Ball SL, Waard JR (2003). Biological identifications through DNA barcodes. Proc. R. Soc. Lond. B Biol. Sci..

[2] Kress WJ, Wurdack KJ, Zimmer EA, Weigt LA, Janzen DH (2005). Use of DNA barcodes to identify flowering plants. Proc. Natl. Acad. Sci. U.S.A..

[3] Kress WJ (2009). Plant DNA barcodes and a community phylogeny of a tropical forest dynamics plot in Panama. Proc. Natl. Acad. Sci. U.S.A..

[4] CBOL Plant Working Group (2009). A DNA barcode for land plants. Proc. Natl. Acad. Sci. U.S.A..

[5] Li XW (2015). Plant DNA barcoding: from gene to genome. Biol. Rev. Camb. Philos. Soc..

[6] Hollingsworth PM, Graham SW, Little DP (2011). Choosing and using a plant DNA barcode. PLoS ONE.

[7] Ahmed I (2013). Identification of chloroplast genome loci suitable for highresolution phylogeographic studies of *Colocasia**esculenta* (L.) Schott (Araceae) and closely related taxa. Mol. Ecol. Resour..

[8] Kuang DY (2011). Complete chloroplast genome sequence of *Magnolia kwangsiensis* (Magnoliaceae): implication for DNA barcoding and population genetics. Genome.

[9] Dong W, Liu J, Yu J, Wang L, Zhou S (2012). Highly variable chloroplast markers for evaluating plant phylogeny at low taxonomic levels and for DNA barcoding. PLoS ONE.

[10] Tamura, M. Ranunculaceae. in *Die natürlichen pflanzenfamilien*, Aufl. II. 2nd ed. 17a IV (ed Hiepko, P.) (Duncker und Humblot, Berlin, 1995).

[11] Wang, W. C. *et al. Flora of China* Vol. 6, 133–438 (Science Press, Beijing; Missouri Botanical Garden Press, St. Louis, 2001).

[12] Grey-Wilson C (2014). Pasque-flowers. The Genus Pulsatilla.

[13] Ren Y, Gu TQ, Chang HL (2011). Floral development of *Dichocarpum*, *Thalictrum*, and *Aquilegia* (Thalictroideae, Ranunculaceae). Plant Syst. Evol..

[14] Ren Y, Chang HL, Endress PK (2015). Floral development in Anemoneae (Ranunculaceae). Bot. J. Linn. Soc..

[15] Li QJ (2019). Efficient identification of *Pulsatilla* (Ranunculaceae) using DNA barcodes and micro-morphological characters. Front. Plant Sci..

[16] China Pharmacopoeia Committee (2015). Pharmacopoeia of the People’s Republic of China.

[17] Xu Q (2012). Antitumor activity of *Pulsatilla chinensis* (Bunge) Regel saponins in human liver tumor 7402 cells in vitro and in vivo. Phytomedicine.

[18] Wang XW, Fan FG, Cao Q (2016). Modified *Pulsatilla* decoction attenuates oxazolone-induced colitis in mice through suppression of inflammation and epithelial barrier disruption. Mol. Med. Rep..

[19] Suh SY, An WG (2017). Systems pharmacological approach of *Pulsatillae* radix on treating Crohn's disease. Evid. Based Complement. Altern. Med..

[20] Szczecińska M, Sawicki J (2015). Genomic resources of three *Pulsatilla* species reveal evolutionary hotspots, species-specific sites and variable plastid structure in the family Ranunculaceae. Int. J. Mol. Sci..

[21] Hoot SB, Reznicek AA, Palmer JD (1994). Phylogenetic relationship in *Anemone* (Ranunculaceae) based on morphology and chloroplast DNA. Syst. Bot..

[22] Hoot SB, Jensen U, Kadereit JW (1995). Phylogenetic relationships in *Anemone* (Ranunculaceae) based on DNA restriction site variation and morphology. Systematics and Evolution of the Ranunculiflorae.

[23] Szczecińska M, Gabor S, Katarzyna W, Jakub S, Dusan G (2016). Genetic diversity and population structure of the rare and endangered plant species *Pulsatilla**patens* (L.) Mill in east central Europe. PLoS ONE.

[24] Jiang N (2017). Phylogenetic reassessment of tribe Anemoneae (Ranunculaceae): non-monophyly of *Anemone* s.l. revealed by plastid datasets. PLoS ONE.

[25] Sramkó G, Laczkó L, Volkova PA, Bateman RM, Mlinarec J (2019). Evolutionary history of the Pasque-flowers (*Pulsatilla*, Ranunculaceae): molecular phylogenetics, systematics and rDNA evolution. Mol. Phylogenet. Evol..

[26] Yu XQ, Yang D, Guo C, Gao LM (2018). Plant phylogenomics based on genome-partitioning strategies: progress and prospects. Plant Divers..

[27] Tang Y, Yukawa T, Bateman RM, Jiang H, Peng H (2015). Phylogeny and classification of the East Asian *Amitostigma alliance* (Orchidaceae: Orchideae) based on six DNA markers. BMC Evol. Biol..

[28] Henriquez CL (2020). Molecular evolution of chloroplast genomes in Monsteroideae (Araceae). Planta.

[29] Abdullah, *et al.* Correlations among oligonucleotide repeats, nucleotide substitutions, and insertion–deletion mutations in chloroplast genomes of plant family Malvaceae. *J. Syst. Evol.*10.1111/jse.12585 (2020).

[30] Abdullah, *et al.* Chloroplast genome of *Hibiscus rosa-sinensis* (Malvaceae): comparative analyses and identification of mutational hotspots. *Genomics* **112**, 581–591 (2020). 10.1016/j.ygeno.2019.04.010 30998967

[31] Liu E (2019). Comparative analysis of complete chloroplast genome sequences of four major *Amorphophallus* species. Sci. Rep..

[32] Chumley, T. W. *et al.* The complete chloroplast genome sequence of *Pelargonium* × *hortorum*: organization and evolution of the largest and most highly rearranged chloroplast genome of land plants. *Mol. Biol. Evol.* **23**, 2175–2190. 10.1093/molbev/msl089 16916942

[33] Ogihara Y (2002). Structural features of a wheat plastome as revealed by complete sequencing of chloroplast DNA. Mol. Genet. Genomics.

[34] Raman G, Park S (2016). The complete chloroplast genome sequence of *Ampelopsis*: gene organization, comparative analysis, and phylogenetic relationships to other angiosperms. Front. Plant Sci..

[35] Daniell H, Lin CS, Yu M, Chang WJ (2016). Chloroplast genomes: diversity, evolution, and applications in genetic engineering. Genome Biol..

[36] Henriquez CL (2020). Evolutionary dynamics in chloroplast genomes of subfamily Aroideae (Araceae). Genomics.

[37] Wang W, Messing J (2011). High-throughput sequencing of three Lemnoideae (duckweeds) chloroplast genomes from total DNA. PLoS ONE.

[38] Adbulch (2020). Complete chloroplast genomes of *Anthurium huixtlense* and *Pothos scandens* (Pothoideae, Araceae): unique inverted repeat expansion and contraction affect rate of evolution. J. Mol. Evol..

[39] Oldenburg DJ, Bendich AJ (2016). The linear plastid chromosomes of maize: terminal sequences, structures, and implications for DNA replication. Curr. Genet..

[40] Sun JH (2020). Evolutionary and phylogenetic aspects of the chloroplast genome of *Chaenomeles* species. Sci. Rep..

[41] Liu HJ (2018). Comparative analysis of complete chloroplast genomes of *Anemoclema*, *Anemone*, *Pulsatilla*, and *Hepatica* revealing structural variations among genera in tribe Anemoneae (Ranunculaceae). Front. Plant Sci..

[42] Meng J (2018). Comparative analysis of the complete chloroplast genomes of four *Aconitum* medicinal species. Molecules.

[43] Du YP (2017). Complete chloroplast genome sequences of *Lilium*: insights into evolutionary dynamics and phylogenetic analyses. Sci. Rep..

[44] Bi Y, Zhang MF, Xue J, Dong R, Du YP, Zhang XH (2018). Chloroplast genomic resources for phylogeny and DNA barcoding: a case study on *Fritillaria*. Sci. Rep..

[45] Choi KS, Chung MG, Park S (2016). The complete chloroplast genome sequences of three Veroniceae species (Plantaginaceae): comparative analysis and highly divergent regions. Front. Plant Sci..

[46] Menezes APA (2018). Chloroplast genomes of *Byrsonima* species (Malpighiaceae): Comparative analysis and screening of high divergence sequences. Sci. Rep..

[47] Li Y, Zhang Z, Yang J, Lv G (2018). Complete chloroplast genome of seven *Fritillaria* species, variable DNA markers identification and phylogenetic relationships within the genus. PLoS ONE.

[48] Grassi F, Labra M, Scienza A, Imazio S (2002). Chloroplast SSR markers to assess DNA diversity in wild and cultivated grapevines. Vitis.

[49] Chen C, Zhou P, Choi YA, Huang S, Gmitter FG (2006). Mining and characterizing microsatellites from citrus ESTs. Theor. Appl. Genet..

[50] Powell W, Morgante M, McDevitt R, Vendramin GG, Rafalski JA (1995). Polymorphic simple sequence repeat regions in chloroplast genomes: applications to the population genetics of pines. Proc. Natl. Acad. Sci. U.S.A..

[51] Kaundun SS, Matsumoto S (2002). Heterologous nuclear and chloroplast microsatellite amplification and variation in tea, *Camellia sinensis*. Genome.

[52] Doorduin L (2011). The complete chloroplast genome of 17 individuals of pest species *Jacobaea vulgaris*: SNPs, microsatellites and barcoding markers for population and phylogenetic studies. DNA Res..

[53] Jiao Y (2012). Development of simple sequence repeat (SSR) markers from a genome survey of Chinese bayberry (*Myrica rubra*). BMC Genomics.

[54] He S, Wang Y, Volis S, Li D, Yi T (2012). Genetic diversity and population structure: implications for conservation of wild soybean (*Glycine**soja* Sieb. et Zucc) based on nuclear and chloroplast microsatellite variation. Int. J. Mol. Sci..

[55] Zhang N (2017). An analysis of *Echinacea* chloroplast genomes: implications for future botanical identification. Sci. Rep..

[56] Li HT (2019). Origin of angiosperms and the puzzle of the Jurassic gap. Nat. Plants.

[57] Kearse M (2012). Geneious Basic: an integrated and extendable desktop software platform for the organization and analysis of sequence data. Bioinformatics.

[58] Qu XJ, Moore MJ, Li DZ, Yi TS (2019). PGA: a software package for rapid, accurate, and flexible batch annotation of plastomes. Plant Methods.

[59] Tillich M (2017). GeSeq-versatile and accurate annotation of organelle genomes. Nucleic Acids Res..

[60] Lowe TM, Chen PP (2016). tRNAscan-SE On-line: integrating search and context for analysis of transfer RNA genes. Nucleic Acids Res..

[61] Lohse M, Drechsel O, Bock R (2007). OrganellarGenomeDRAW (OGDRAW): a tool for the easy generation of high-quality custom graphical maps of plastid and mitochondrial genomes. Curr. Genet..

[62] Katoh K, Standley DM (2013). MAFFT multiple sequence alignment software version 7: improvements in performance and usability. Mol. Biol. Evol..

[63] Amiryousefi A, Hyvönen J, Poczai P (2018). IRscope: an online program to visualize the junction sites of chloroplast genomes. Bioinformatics.

[64] Frazer KA, Pachter L, Poliakov A, Rubin EM, Dubchak I (2004). VISTA: computational tools for comparative genomics. Nucleic Acids. Res..

[65] Darling AC, Mau B, Blattner FR, Perna NT (2004). Mauve: multiple alignment of conserved genomic sequence with rearrangements. Genome Res..

[66] Librado P, Rozas J (2009). DnaSP v5: a software for comprehensive analysis of DNA polymorphism data. Bioinformatics.

[67] Kurtz S (2001). REPuter: the manifold applications of repeat analysis on a genomic scale. Nucleic Acids. Res..

[68] Thiel T, Michalek W, Varshney R, Graner A (2003). Exploiting EST databases for the development and characterization of gene-derived SSR markers in barley (*Hordeum**vulgare* L.). Theor. Appl. Genet..

[69] Stamatakis A (2014). RAxML version 8: A tool for phylogenetic analysis and post-analysis of large phylogenies. Bioinformatics.

[70] Miller, M. A., Pfeiffer, W. & Schwartz, T. Creating the CIPRES science gateway for inference of large phylogenetic trees. New Orleans, LA. *Proceedings of the Gateway Computing Environments Workshop *(*GCE*), pp. 1–8 (2010).

[71] Darriba D, Taboada GL, Doallo R, Posada D (2012). jModelTest 2: more models, new heuristics and parallel computing. Nat. Methods.

[72] Swofford, D. L. PAUP*: phylogenetic analysis using Parsimony (*and other methods), version 4.0b10 (Sinauer Associates, Sunderland, 2003).

