# Supplementary figures and images for: Chloroplast genomes elucidate diversity, phylogeny, and taxonomy of Pulsatilla (Ranunculaceae)
Source: Sci Rep. 2020 Nov 13;10:19781. doi: 10.1038/s41598-020-76699-7 (PMC7666119; doi:10.1038/s41598-020-76699-7)

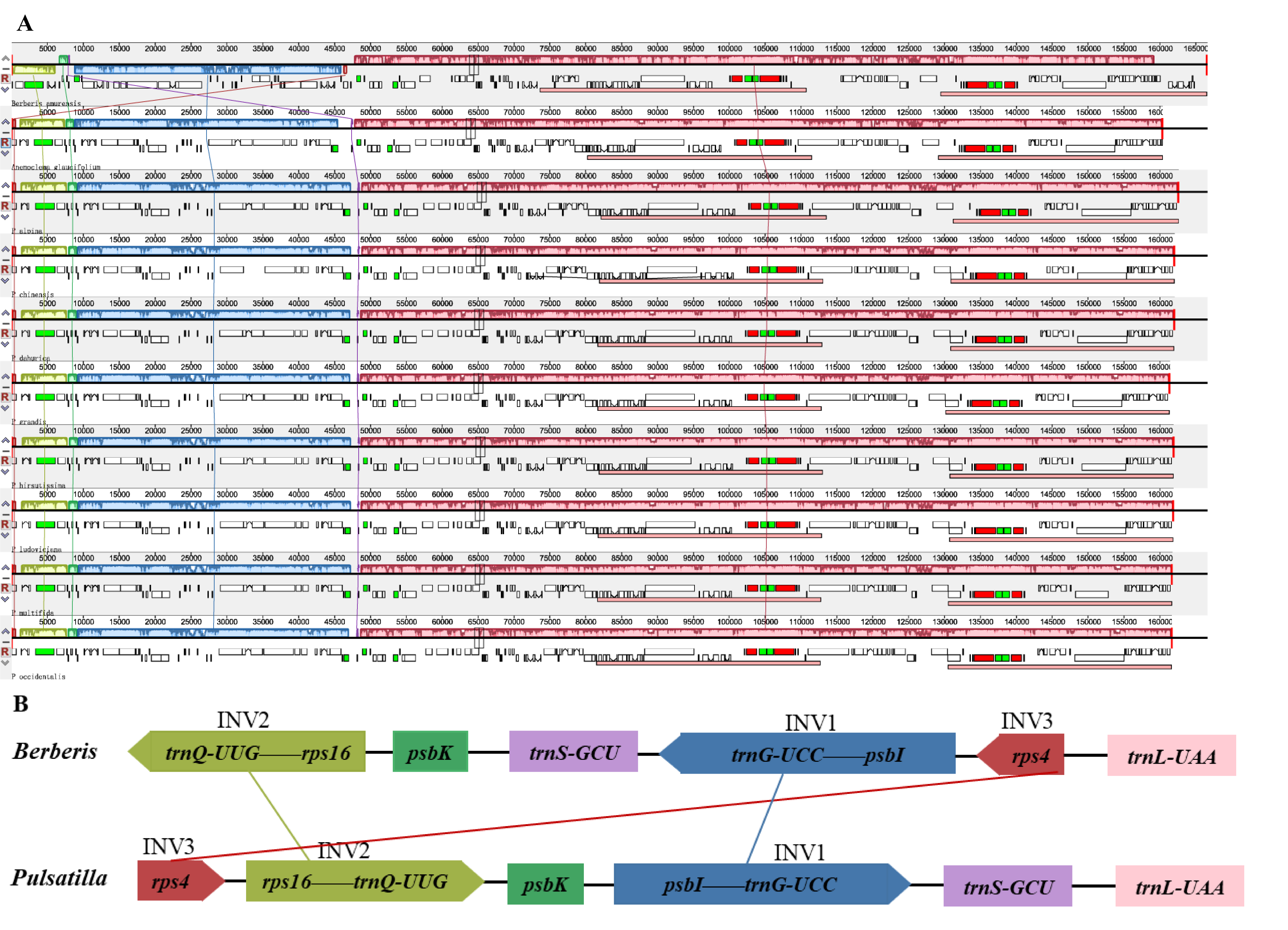

Supplement: Supplementary file 1 — Supplementary Figure S1. [file 41598_2020_76699_MOESM1_ESM.tif]
