# Supplementary material for: Chloroplast genomes elucidate diversity, phylogeny, and taxonomy of Pulsatilla (Ranunculaceae)
Source: Sci Rep. 2020 Nov 13;10:19781. doi: 10.1038/s41598-020-76699-7 (PMC7666119; doi:10.1038/s41598-020-76699-7)
Supplement: Supplementary file 2 — Supplementary Table S1. [file 41598_2020_76699_MOESM2_ESM.doc]

**Table S1.** Rate of synonymous and non-synonymous substitutions

| Gene | species | synDif | SynPos | Ks | NsynDif | NSynPos | Ka |
| --- | --- | --- | --- | --- | --- | --- | --- |
| *rps4* | *alpina* | 7.83 | 141.25 | 0.0576 | 14.17 | 446.75 | 0.0324 |
|  | *dahurica* | 0 | 142.33 | 0 | 0 | 445.67 | 0 |
|  | *grandis* | 0 | 142.33 | 0 | 0 | 445.67 | 0 |
|  | *hirsutissima* | 0 | 142.33 | 0 | 0 | 445.67 | 0 |
|  | *ludoviciana* | 0 | 142.33 | 0 | 0 | 445.67 | 0 |
|  | *multifida* | 0 | 123.33 | 0 | 1 | 461.67 | 0.0022 |
|  | *occidentalis* | 2 | 141.92 | 0.0142 | 2 | 446.08 | 0.0045 |
| *rps16* | *alpina* | 2 | 198.67 | 0.0101 | 8 | 788.33 | 0.0102 |
|  | *dahurica* | 0 | 213.67 | 0 | 4 | 806.33 | 0.0050 |
|  | *grandis* | 0 | 244.92 | 0 | 7 | 865.08 | 0.0081 |
|  | *hirsutissima* | 1 | 229.67 | 0.0044 | 7 | 871.33 | 0.0081 |
|  | *ludoviciana* | 1 | 229.67 | 0.0044 | 7 | 871.33 | 0.0081 |
|  | *multifida* | 0 | 221.58 | 0 | 8 | 882.42 | 0.0091 |
|  | *occidentalis* | 3.17 | 220.50 | 0.0145 | 16.83 | 868.50 | 0.0196 |
| *matK* | *alpina* | 5 | 342.92 | 0.0147 | 3 | 1181.08 | 0.0025 |
|  | *dahurica* | 1 | 343.08 | 0.0029 | 2 | 1180.92 | 0.0017 |
|  | *grandis* | 0 | 343.08 | 0 | 2 | 1180.92 | 0.0017 |
|  | *hirsutissima* | 1 | 342.92 | 0.0029 | 3 | 1181.08 | 0.0025 |
|  | *ludoviciana* | 1 | 342.92 | 0.0029 | 3 | 1181.08 | 0.0025 |
|  | *multifida* | 1 | 342.92 | 0.0029 | 3 | 1181.08 | 0.0025 |
|  | *occidentalis* | 3 | 342.75 | 0.0088 | 9 | 1181.25 | 0.0077 |
| *psbA* | *alpina* | 1 | 250.33 | 0.0040 | 0 | 808.67 | 0 |
|  | *dahurica* | 2 | 250.17 | 0.0080 | 0 | 808.83 | 0 |
|  | *grandis* | 1 | 250.33 | 0.0040 | 0 | 808.67 | 0 |
|  | *hirsutissima* | 1 | 250.33 | 0.0040 | 0 | 808.67 | 0 |
|  | *ludoviciana* | 1 | 250.33 | 0.0040 | 0 | 808.67 | 0 |
|  | *multifida* | 1 | 250.33 | 0.0040 | 0 | 808.67 | 0 |
|  | *occidentalis* | 1 | 250.33 | 0.0040 | 0 | 808.67 | 0 |
| *psbK* | *alpina* | 0 | 39.33 | 0 | 0 | 143.67 | 0 |
|  | *dahurica* | 0 | 39.33 | 0 | 0 | 143.67 | 0 |
|  | *grandis* | 0 | 39.33 | 0 | 0 | 143.67 | 0 |
|  | *hirsutissima* | 0 | 39.17 | 0 | 1 | 143.83 | 0.0070 |
|  | *ludoviciana* | 0 | 39.17 | 0 | 1 | 143.83 | 0.0070 |
|  | *multifida* | 0 | 39.33 | 0 | 0 | 143.67 | 0 |
|  | *occidentalis* | 0 | 39.33 | 0 | 0 | 143.67 | 0 |
| *psbI* | *alpina* | 0 | 26.67 | 0 | 0 | 81.33 | 0 |
|  | *dahurica* | 0 | 26.67 | 0 | 0 | 81.33 | 0 |
|  | *grandis* | 0 | 26.67 | 0 | 0 | 81.33 | 0 |
|  | *hirsutissima* | 0 | 26.67 | 0 | 0 | 81.33 | 0 |
|  | *ludoviciana* | 0 | 26.67 | 0 | 0 | 81.33 | 0 |
|  | *multifida* | 0 | 26.67 | 0 | 0 | 81.33 | 0 |
|  | *occidentalis* | 0 | 26.67 | 0 | 0 | 81.33 | 0 |
| *ycf3* | *alpina* | 1 | 118.67 | 0.0085 | 0 | 385.33 | 0 |
|  | *dahurica* | 1 | 118.67 | 0.0085 | 0 | 385.33 | 0 |
|  | *grandis* | 1 | 118.67 | 0.0085 | 0 | 385.33 | 0 |
|  | *hirsutissima* | 1 | 118.67 | 0.0085 | 0 | 385.33 | 0 |
|  | *ludoviciana* | 1 | 118.67 | 0.0085 | 0 | 385.33 | 0 |
|  | *multifida* | 1 | 118.67 | 0.0085 | 0 | 385.33 | 0 |
|  | *occidentalis* | 1 | 118.67 | 0.0085 | 0 | 385.33 | 0 |
| *psaA* | *alpina* | 6 | 451.83 | 0.0134 | 5 | 1483.17 | 0.0034 |
|  | *dahurica* | 2 | 450.17 | 0.0045 | 2 | 1478.83 | 0.0014 |
|  | *grandis* | 3 | 451.83 | 0.0067 | 5 | 1483.17 | 0.0034 |
|  | *hirsutissima* | 3 | 452.17 | 0.0067 | 5 | 1482.83 | 0.0034 |
|  | *ludoviciana* | 3 | 452.17 | 0.0067 | 5 | 1482.83 | 0.0034 |
|  | *multifida* | 3 | 452.17 | 0.0067 | 5 | 1482.83 | 0.0034 |
|  | *occidentalis* | 5 | 452.00 | 0.0111 | 5 | 1483.00 | 0.0034 |
| *psaB* | *alpina* | 0 | 496.50 | 0 | 3 | 1702.50 | 0.0018 |
|  | *dahurica* | 0 | 498.83 | 0 | 1 | 1709.17 | 0.0006 |
|  | *grandis* | 0 | 496.17 | 0 | 1 | 1702.83 | 0.0006 |
|  | *hirsutissima* | 0 | 496.33 | 0 | 2 | 1702.67 | 0.0012 |
|  | *ludoviciana* | 0 | 496.33 | 0 | 2 | 1702.67 | 0.0012 |
|  | *multifida* | 0 | 496.50 | 0 | 1 | 1702.50 | 0.0006 |
|  | *occidentalis* | 0 | 496.42 | 0 | 3 | 1702.58 | 0.0018 |
| *rps14* | *alpina* | 0 | 68.50 | 0 | 1 | 231.50 | 0.0043 |
|  | *dahurica* | 0 | 68.50 | 0 | 0 | 231.50 | 0 |
|  | *grandis* | 0 | 68.50 | 0 | 1 | 231.50 | 0.0043 |
|  | *hirsutissima* | 0 | 68.50 | 0 | 1 | 231.50 | 0.0043 |
|  | *ludoviciana* | 0 | 68.50 | 0 | 1 | 231.50 | 0.0043 |
|  | *multifida* | 0 | 68.50 | 0 | 1 | 231.50 | 0.0043 |
|  | *occidentalis* | 0 | 68.50 | 0 | 1 | 231.50 | 0.0043 |
| *psbZ* | *alpina* | 0 | 46.33 | 0 | 0 | 139.67 | 0 |
|  | *dahurica* | 0 | 46.33 | 0 | 0 | 139.67 | 0 |
|  | *grandis* | 0 | 46.33 | 0 | 0 | 139.67 | 0 |
|  | *hirsutissima* | 0 | 46.33 | 0 | 0 | 139.67 | 0 |
|  | *ludoviciana* | 0 | 46.33 | 0 | 0 | 139.67 | 0 |
|  | *multifida* | 0 | 46.33 | 0 | 0 | 139.67 | 0 |
|  | *occidentalis* | 0 | 46.33 | 0 | 0 | 139.67 | 0 |
| *psbC* | *alpina* | 1 | 350.50 | 0.0029 | 0 | 1068.50 | 0 |
|  | *dahurica* | 2 | 350.25 | 0.0057 | 1 | 1068.75 | 0.0009 |
|  | *grandis* | 0 | 350.50 | 0 | 0 | 1068.50 | 0 |
|  | *hirsutissima* | 0 | 350.50 | 0 | 0 | 1068.50 | 0 |
|  | *ludoviciana* | 0 | 350.50 | 0 | 0 | 1068.50 | 0 |
|  | *multifida* | 0 | 350.50 | 0 | 0 | 1068.50 | 0 |
|  | *occidentalis* | 0 | 350.50 | 0 | 0 | 1068.50 | 0 |
| *psbD* | *alpina* | 1 | 252.17 | 0.0040 | 0 | 806.83 | 0 |
|  | *dahurica* | 0 | 252.17 | 0 | 0 | 806.83 | 0 |
|  | *grandis* | 0 | 252.17 | 0 | 0 | 806.83 | 0 |
|  | *hirsutissima* | 0 | 252.17 | 0 | 0 | 806.83 | 0 |
|  | *ludoviciana* | 0 | 252.17 | 0 | 0 | 806.83 | 0 |
|  | *multifida* | 0 | 252.17 | 0 | 0 | 806.83 | 0 |
|  | *occidentalis* | 1 | 252.17 | 0.0040 | 0 | 806.83 | 0 |
| *psbM* | *alpina* | 0 | 25 | 0 | 0 | 77 | 0 |
|  | *dahurica* | 0 | 25 | 0 | 0 | 77 | 0 |
|  | *grandis* | 0 | 25 | 0 | 0 | 77 | 0 |
|  | *hirsutissima* | 0 | 25 | 0 | 0 | 77 | 0 |
|  | *ludoviciana* | 0 | 25 | 0 | 0 | 77 | 0 |
|  | *multifida* | 0 | 25 | 0 | 0 | 77 | 0 |
|  | *occidentalis* | 0 | 25 | 0 | 0 | 77 | 0 |
| *petN* | *alpina* | 0 | 20.83 | 0 | 0 | 66.17 | 0 |
|  | *dahurica* | 0 | 20.83 | 0 | 0 | 66.17 | 0 |
|  | *grandis* | 0 | 20.83 | 0 | 0 | 66.17 | 0 |
|  | *hirsutissima* | 0 | 20.83 | 0 | 0 | 66.17 | 0 |
|  | *ludoviciana* | 0 | 20.83 | 0 | 0 | 66.17 | 0 |
|  | *multifida* | 0 | 20.83 | 0 | 0 | 66.17 | 0 |
|  | *occidentalis* | 0 | 20.83 | 0 | 0 | 66.17 | 0 |
| *rpoB* | *alpina* | 5 | 758.33 | 0.0066 | 3 | 2451.67 | 0.0012 |
|  | *dahurica* | 0 | 758.33 | 0 | 0 | 2451.67 | 0 |
|  | *grandis* | 1 | 758.33 | 0.0013 | 1 | 2451.67 | 0.0004 |
|  | *hirsutissima* | 1 | 758.33 | 0.0013 | 1 | 2451.67 | 0.0004 |
|  | *ludoviciana* | 1 | 758.33 | 0.0013 | 2 | 2451.67 | 0.0008 |
|  | *multifida* | 1 | 758.33 | 0.0013 | 1 | 2451.67 | 0.0004 |
|  | *occidentalis* | 5 | 758.33 | 0.0066 | 4 | 2451.67 | 0.0016 |
| *rpoC1* | *alpina* | 3 | 479.33 | 0.0063 | 2 | 1578.67 | 0.0013 |
|  | *dahurica* | 1 | 479.67 | 0.0021 | 0 | 1588.33 | 0 |
|  | *grandis* | 1 | 479.33 | 0.0021 | 0 | 1578.67 | 0 |
|  | *hirsutissima* | 1 | 479.67 | 0.0021 | 0 | 1584.33 | 0 |
|  | *ludoviciana* | 1 | 479.67 | 0.0021 | 1 | 1584.33 | 0.0006 |
|  | *multifida* | 1 | 479.67 | 0.0021 | 0 | 1584.33 | 0 |
|  | *occidentalis* | 5 | 479.33 | 0.0105 | 3 | 1578.67 | 0.0019 |
| *rpoC2* | *alpina* | 12 | 970.25 | 0.0125 | 8 | 3178.75 | 0.0025 |
|  | *dahurica* | 7 | 968.92 | 0.0073 | 5 | 3180.08 | 0.0016 |
|  | *grandis* | 5 | 969.75 | 0.0052 | 3 | 3179.25 | 0.0009 |
|  | *hirsutissima* | 6 | 969.83 | 0.0062 | 3 | 3179.17 | 0.0009 |
|  | *ludoviciana* | 6 | 969.83 | 0.0062 | 3 | 3179.17 | 0.0009 |
|  | *multifida* | 7 | 969.83 | 0.0073 | 4 | 3179.17 | 0.0013 |
|  | *occidentalis* | 9 | 970.50 | 0.0093 | 9 | 3178.50 | 0.0028 |
| *rps2* | *alpina* | 2 | 164.17 | 0.0123 | 0 | 540.83 | 0 |
|  | *dahurica* | 2 | 164.17 | 0.0123 | 0 | 540.83 | 0 |
|  | *grandis* | 2 | 164.17 | 0.0123 | 0 | 540.83 | 0 |
|  | *hirsutissima* | 2 | 164.17 | 0.0123 | 0 | 540.83 | 0 |
|  | *ludoviciana* | 2 | 164.17 | 0.0123 | 0 | 540.83 | 0 |
|  | *multifida* | 2 | 164.17 | 0.0123 | 0 | 540.83 | 0 |
|  | *occidentalis* | 2 | 164.17 | 0.0123 | 0 | 540.83 | 0 |
| *atpI* | *alpina* | 0 | 179.50 | 0 | 0 | 561.50 | 0 |
|  | *dahurica* | 0 | 179.50 | 0 | 0 | 561.50 | 0 |
|  | *grandis* | 0 | 179.50 | 0 | 0 | 561.50 | 0 |
|  | *hirsutissima* | 0 | 179.50 | 0 | 0 | 561.50 | 0 |
|  | *ludoviciana* | 0 | 179.50 | 0 | 0 | 561.50 | 0 |
|  | *multifida* | 0 | 179.50 | 0 | 0 | 561.50 | 0 |
|  | *occidentalis* | 0 | 179.50 | 0 | 0 | 561.50 | 0 |
| *atpH* | *alpina* | 0 | 67.33 | 0 | 0 | 175.67 | 0 |
|  | *dahurica* | 0 | 67.33 | 0 | 0 | 175.67 | 0 |
|  | *grandis* | 0 | 67.33 | 0 | 0 | 175.67 | 0 |
|  | *hirsutissima* | 0 | 67.33 | 0 | 0 | 175.67 | 0 |
|  | *ludoviciana* | 1 | 67.33 | 0.0150 | 0 | 175.67 | 0 |
|  | *multifida* | 0 | 67.33 | 0 | 0 | 175.67 | 0 |
|  | *occidentalis* | 0 | 67.33 | 0 | 0 | 175.67 | 0 |
| *atpF* | *alpina* | 1 | 123.67 | 0.0081 | 2 | 428.33 | 0.0047 |
|  | *dahurica* | 1 | 123.67 | 0.0081 | 1 | 428.50 | 0.0023 |
|  | *grandis* | 0 | 123.33 | 0 | 2 | 428.67 | 0.0047 |
|  | *hirsutissima* | 1 | 123.67 | 0.0081 | 1 | 428.50 | 0.0023 |
|  | *ludoviciana* | 1 | 123.67 | 0.0081 | 1 | 428.50 | 0.0023 |
|  | *multifida* | 1 | 123.67 | 0.0081 | 1 | 428.50 | 0.0023 |
|  | *occidentalis* | 1 | 123.67 | 0.0081 | 2 | 428.33 | 0.0047 |
| *atpA* | *alpina* | 3 | 372.67 | 0.0081 | 0 | 1148.33 | 0 |
|  | *dahurica* | 0 | 372.67 | 0 | 1 | 1148.33 | 0.0009 |
|  | *grandis* | 0 | 372.67 | 0 | 0 | 1148.33 | 0 |
|  | *hirsutissima* | 0 | 372.67 | 0 | 0 | 1148.33 | 0 |
|  | *ludoviciana* | 0 | 372.67 | 0 | 0 | 1148.33 | 0 |
|  | *multifida* | 0 | 372.67 | 0 | 0 | 1148.33 | 0 |
|  | *occidentalis* | 3 | 372.67 | 0.0081 | 0 | 1148.33 | 0 |
| *ndhJ* | *alpina* | 0 | 110.00 | 0 | 0 | 364.00 | 0 |
|  | *dahurica* | 0 | 110.00 | 0 | 0 | 364.00 | 0 |
|  | *grandis* | 0 | 110.00 | 0 | 0 | 364.00 | 0 |
|  | *hirsutissima* | 0 | 110.00 | 0 | 0 | 364.00 | 0 |
|  | *ludoviciana* | 0 | 110.00 | 0 | 0 | 364.00 | 0 |
|  | *multifida* | 0 | 110.00 | 0 | 0 | 364.00 | 0 |
|  | *occidentalis* | 0 | 110.00 | 0 | 0 | 364.00 | 0 |
| *ndhK* | *alpina* | 2 | 165.67 | 0.0122 | 3 | 515.33 | 0.0058 |
|  | *dahurica* | 0 | 165.67 | 0 | 4 | 515.33 | 0.0078 |
|  | *grandis* | 0 | 156.33 | 0 | 2 | 485.67 | 0.0041 |
|  | *hirsutissima* | 0 | 165.67 | 0 | 2 | 515.33 | 0.0039 |
|  | *ludoviciana* | 0 | 165.67 | 0 | 2 | 515.33 | 0.0039 |
|  | *multifida* | 0 | 156.33 | 0 | 2 | 485.67 | 0.0041 |
|  | *occidentalis* | 1 | 165.50 | 0.0061 | 3 | 515.50 | 0.0058 |
| *ndhC* | *alpina* | 1 | 84.17 | 0.0120 | 0 | 275.83 | 0 |
|  | *dahurica* | 0 | 84.17 | 0 | 0 | 275.83 | 0 |
|  | *grandis* | 1 | 84.17 | 0.0120 | 0 | 275.83 | 0 |
|  | *hirsutissima* | 1 | 84.42 | 0.0119 | 1 | 275.58 | 0.0036 |
|  | *ludoviciana* | 1 | 84.42 | 0.0119 | 1 | 275.58 | 0.0036 |
|  | *multifida* | 1 | 84.42 | 0.0119 | 1 | 275.58 | 0.0036 |
|  | *occidentalis* | 1 | 84.17 | 0.0120 | 0 | 275.83 | 0 |
| *atpE* | *alpina* | 0 | 94.00 | 0 | 0 | 305.00 | 0 |
|  | *dahurica* | 1 | 94.00 | 0.0107 | 0 | 305.00 | 0 |
|  | *grandis* | 0 | 94.00 | 0 | 0 | 305.00 | 0 |
|  | *hirsutissima* | 0 | 94.00 | 0 | 0 | 305.00 | 0 |
|  | *ludoviciana* | 0 | 94.00 | 0 | 0 | 305.00 | 0 |
|  | *multifida* | 0 | 94.00 | 0 | 0 | 305.00 | 0 |
|  | *occidentalis* | 1 | 94.00 | 0.0107 | 0 | 305.00 | 0 |
| *atpB* | *alpina* | 4 | 373.00 | 0.0108 | 0 | 1127.00 | 0 |
|  | *dahurica* | 2 | 373.00 | 0.0054 | 0 | 1127.00 | 0 |
|  | *grandis* | 2 | 373.00 | 0.0054 | 0 | 1127.00 | 0 |
|  | *hirsutissima* | 2 | 373.00 | 0.0054 | 0 | 1127.00 | 0 |
|  | *ludoviciana* | 2 | 373.00 | 0.0054 | 0 | 1127.00 | 0 |
|  | *multifida* | 2 | 373.00 | 0.0054 | 0 | 1127.00 | 0 |
|  | *occidentalis* | 3 | 373.00 | 0.0081 | 1 | 1127.00 | 0.0009 |
| *rbcL* | *alpina* | 2.5 | 342.83 | 0.0073 | 7.5 | 1082.17 | 0.0070 |
|  | *dahurica* | 0 | 342.50 | 0 | 2 | 1082.50 | 0.0018 |
|  | *grandis* | 0 | 342.50 | 0 | 2 | 1082.50 | 0.0018 |
|  | *hirsutissima* | 0 | 343.17 | 0 | 5 | 1081.83 | 0.0046 |
|  | *ludoviciana* | 0 | 343.17 | 0 | 5 | 1081.83 | 0.0046 |
|  | *multifida* | 0 | 343.17 | 0 | 5 | 1081.83 | 0.0046 |
|  | *occidentalis* | 1.5 | 324.08 | 0.0046 | 10.5 | 1028.92 | 0.0103 |
| *accD* | *alpina* | 0 | 303.33 | 0 | 2 | 1139.67 | 0.0018 |
|  | *dahurica* | 2 | 303.50 | 0.0066 | 1 | 1139.50 | 0.0009 |
|  | *grandis* | 1 | 303.50 | 0.0033 | 1 | 1139.50 | 0.0009 |
|  | *hirsutissima* | 1 | 303.42 | 0.0033 | 1 | 1139.52 | 0.0009 |
|  | *ludoviciana* | 1 | 303.42 | 0.0033 | 1 | 1139.52 | 0.0009 |
|  | *multifida* | 0 | 303.50 | 0 | 1 | 1139.50 | 0.0009 |
|  | *occidentalis* | 0 | 303.50 | 0 | 1 | 1139.50 | 0.0009 |
| *psaI* | *alpina* | 0 | 26.33 | 0 | 0 | 81.67 | 0 |
|  | *dahurica* | 0 | 26.33 | 0 | 0 | 81.67 | 0 |
|  | *grandis* | 0 | 26.33 | 0 | 0 | 81.67 | 0 |
|  | *hirsutissima* | 0 | 26.33 | 0 | 0 | 81.67 | 0 |
|  | *ludoviciana* | 0 | 26.33 | 0 | 0 | 81.67 | 0 |
|  | *multifida* | 0 | 26.33 | 0 | 0 | 81.67 | 0 |
|  | *occidentalis* | 0 | 26.33 | 0 | 0 | 81.67 | 0 |
| *ycf4* | *alpina* | 0 | 129.00 | 0 | 1 | 423.00 | 0.0024 |
|  | *dahurica* | 0 | 129.17 | 0 | 0 | 422.83 | 0 |
|  | *grandis* | 0 | 129.17 | 0 | 0 | 422.83 | 0 |
|  | *hirsutissima* | 0 | 129.17 | 0 | 0 | 422.83 | 0 |
|  | *ludoviciana* | 0 | 129.17 | 0 | 0 | 422.83 | 0 |
|  | *multifida* | 0 | 129.17 | 0 | 0 | 422.83 | 0 |
|  | *occidentalis* | 0 | 129.00 | 0 | 1 | 423.00 | 0.0024 |
| *cemA* | *alpina* | 3 | 159.33 | 0.0191 | 3 | 593.67 | 0.0051 |
|  | *dahurica* | 1 | 159.33 | 0.0063 | 1 | 593.67 | 0.0017 |
|  | *grandis* | 1 | 159.33 | 0.0063 | 1 | 593.67 | 0.0017 |
|  | *hirsutissima* | 1 | 159.33 | 0.0063 | 1 | 593.67 | 0.0017 |
|  | *ludoviciana* | 1 | 159.33 | 0.0063 | 1 | 593.67 | 0.0017 |
|  | *multifida* | 1 | 159.33 | 0.0063 | 1 | 593.67 | 0.0017 |
|  | *occidentalis* | 3 | 159.33 | 0.0191 | 2 | 594.00 | 0.0034 |
| *petA* | *alpina* | 2 | 227.33 | 0.0088 | 0 | 738.67 | 0 |
|  | *dahurica* | 4 | 227.33 | 0.0133 | 0 | 738.67 | 0 |
|  | *grandis* | 2 | 227.33 | 0.0088 | 0 | 738.67 | 0 |
|  | *hirsutissima* | 2 | 227.33 | 0.0088 | 0 | 738.67 | 0 |
|  | *ludoviciana* | 2 | 227.33 | 0.0088 | 0 | 738.67 | 0 |
|  | *multifida* | 2 | 227.33 | 0.0088 | 0 | 738.67 | 0 |
|  | *occidentalis* | 2 | 227.33 | 0.0088 | 0 | 738.67 | 0 |
| *psbJ* | *alpina* | 0 | 33.67 | 0 | 0 | 86.33 | 0 |
|  | *dahurica* | 0 | 33.67 | 0 | 0 | 86.33 | 0 |
|  | *grandis* | 0 | 33.67 | 0 | 0 | 86.33 | 0 |
|  | *hirsutissima* | 0 | 33.67 | 0 | 0 | 86.33 | 0 |
|  | *ludoviciana* | 0 | 33.67 | 0 | 0 | 86.33 | 0 |
|  | *multifida* | 0 | 33.67 | 0 | 0 | 86.33 | 0 |
|  | *occidentalis* | 0 | 33.67 | 0 | 0 | 86.33 | 0 |
| *psbL* | *alpina* | 0 | 24.33 | 0 | 0 | 89.67 | 0 |
|  | *dahurica* | 0 | 24.33 | 0 | 0 | 89.67 | 0 |
|  | *grandis* | 0 | 24.33 | 0 | 0 | 89.67 | 0 |
|  | *hirsutissima* | 0 | 24.33 | 0 | 0 | 89.67 | 0 |
|  | *ludoviciana* | 0 | 24.33 | 0 | 0 | 89.67 | 0 |
|  | *multifida* | 0 | 24.33 | 0 | 0 | 89.67 | 0 |
|  | *occidentalis* | 1 | 24.33 | 0.0423 | 0 | 89.67 | 0 |
| *psbF* | *alpina* | 0 | 30.50 | 0 | 0 | 86.50 | 0 |
|  | *dahurica* | 0 | 30.50 | 0 | 0 | 86.50 | 0 |
|  | *grandis* | 0 | 30.50 | 0 | 0 | 86.50 | 0 |
|  | *hirsutissima* | 0 | 30.50 | 0 | 0 | 86.50 | 0 |
|  | *ludoviciana* | 0 | 30.50 | 0 | 0 | 86.50 | 0 |
|  | *multifida* | 0 | 30.50 | 0 | 0 | 86.50 | 0 |
|  | *occidentalis* | 0 | 30.50 | 0 | 0 | 86.50 | 0 |
| *psbE* | *alpina* | 0 | 58.33 | 0 | 0 | 190.17 | 0 |
|  | *dahurica* | 0 | 58.33 | 0 | 0 | 190.17 | 0 |
|  | *grandis* | 0 | 58.33 | 0 | 0 | 190.17 | 0 |
|  | *hirsutissima* | 0 | 58.33 | 0 | 0 | 190.17 | 0 |
|  | *ludoviciana* | 0 | 58.33 | 0 | 0 | 190.17 | 0 |
|  | *multifida* | 0 | 58.33 | 0 | 0 | 190.17 | 0 |
|  | *occidentalis* | 0 | 58.33 | 0 | 0 | 190.17 | 0 |
| *petL* | *alpina* | 0 | 25.83 | 0 | 0 | 67.17 | 0 |
|  | *dahurica* | 0 | 25.83 | 0 | 0 | 67.17 | 0 |
|  | *grandis* | 0 | 25.83 | 0 | 0 | 67.17 | 0 |
|  | *hirsutissima* | 1 | 25.83 | 0.0397 | 0 | 67.17 | 0 |
|  | *ludoviciana* | 1 | 25.83 | 0.0397 | 0 | 67.17 | 0 |
|  | *multifida* | 1 | 25.83 | 0.0397 | 0 | 67.17 | 0 |
|  | *occidentalis* | 0 | 25.83 | 0 | 0 | 67.17 | 0 |
| *petG* | *alpina* | 0 | 28.83 | 0 | 0 | 82.17 | 0 |
|  | *dahurica* | 0 | 28.83 | 0 | 0 | 82.17 | 0 |
|  | *grandis* | 0 | 28.83 | 0 | 0 | 82.17 | 0 |
|  | *hirsutissima* | 0 | 28.83 | 0 | 0 | 82.17 | 0 |
|  | *ludoviciana* | 0 | 28.83 | 0 | 0 | 82.17 | 0 |
|  | *multifida* | 0 | 28.83 | 0 | 0 | 82.17 | 0 |
|  | *occidentalis* | 0 | 28.83 | 0 | 0 | 82.17 | 0 |
| *psaJ* | *alpina* | 0 | 32.50 | 0 | 0 | 99.50 | 0 |
|  | *dahurica* | 0 | 32.50 | 0 | 0 | 99.50 | 0 |
|  | *grandis* | 0 | 32.50 | 0 | 0 | 99.50 | 0 |
|  | *hirsutissima* | 1 | 32.50 | 0.0314 | 0 | 99.50 | 0 |
|  | *ludoviciana* | 1 | 32.50 | 0.0314 | 0 | 99.50 | 0 |
|  | *multifida* | 1 | 32.50 | 0.0314 | 0 | 99.50 | 0 |
|  | *occidentalis* | 0 | 32.50 | 0 | 0 | 99.50 | 0 |
| *rpl33* | *alpina* | 0 | 42.67 | 0 | 0 | 155.33 | 0 |
|  | *dahurica* | 0 | 42.67 | 0 | 0 | 155.33 | 0 |
|  | *grandis* | 0 | 42.67 | 0 | 0 | 155.33 | 0 |
|  | *hirsutissima* | 0 | 42.67 | 0 | 0 | 155.33 | 0 |
|  | *ludoviciana* | 0 | 42.67 | 0 | 0 | 155.33 | 0 |
|  | *multifida* | 0 | 42.67 | 0 | 0 | 155.33 | 0 |
|  | *occidentalis* | 0 | 42.67 | 0 | 0 | 155.33 | 0 |
| *rps18* | *alpina* | 0 | 72.17 | 0 | 0 | 230.83 | 0 |
|  | *dahurica* | 0 | 72.17 | 0 | 0 | 230.83 | 0 |
|  | *grandis* | 0 | 72.17 | 0 | 0 | 230.83 | 0 |
|  | *hirsutissima* | 0 | 72.17 | 0 | 0 | 230.83 | 0 |
|  | *ludoviciana* | 0 | 72.17 | 0 | 0 | 230.83 | 0 |
|  | *multifida* | 0 | 72.17 | 0 | 0 | 230.83 | 0 |
|  | *occidentalis* | 0 | 72.17 | 0 | 0 | 230.83 | 0 |
| *rpl20* | *alpina* | 0 | 85.00 | 0 | 1 | 266.00 | 0.0038 |
|  | *dahurica* | 0 | 84.67 | 0 | 0 | 266.33 | 0 |
|  | *grandis* | 0 | 84.67 | 0 | 0 | 266.33 | 0 |
|  | *hirsutissima* | 0 | 84.67 | 0 | 0 | 266.33 | 0 |
|  | *ludoviciana* | 0 | 84.67 | 0 | 0 | 266.33 | 0 |
|  | *multifida* | 0 | 84.67 | 0 | 0 | 266.33 | 0 |
|  | *occidentalis* | 0 | 84.92 | 0 | 2 | 266.08 | 0.0076 |
| *rps12* | *alpina* | 0 | 92.67 | 0 | 0 | 271.33 | 0 |
|  | *dahurica* | 0 | 92.67 | 0 | 0 | 271.33 | 0 |
|  | *grandis* | 0 | 92.67 | 0 | 0 | 271.33 | 0 |
|  | *hirsutissima* | 0 | 92.67 | 0 | 0 | 271.33 | 0 |
|  | *ludoviciana* | 0 | 92.67 | 0 | 0 | 252.33 | 0 |
|  | *multifida* | 0 | 92.67 | 0 | 0 | 271.33 | 0 |
|  | *occidentalis* | 0 | 92.67 | 0 | 0 | 271.33 | 0 |
| *clpP* | *alpina* | 3 | 142.33 | 0.0214 | 1 | 457.67 | 0.0022 |
|  | *dahurica* | 1 | 142.00 | 0.0071 | 1 | 458.00 | 0.0022 |
|  | *grandis* | 1 | 142.00 | 0.0071 | 2 | 458.00 | 0.0044 |
|  | *hirsutissima* | 1 | 142.00 | 0.0071 | 1 | 458.00 | 0.0022 |
|  | *ludoviciana* | 1 | 142.00 | 0.0071 | 1 | 458.00 | 0.0022 |
|  | *multifida* | 2 | 142.00 | 0.0142 | 1 | 458.00 | 0.0022 |
|  | *occidentalis* | 1 | 142.00 | 0.0071 | 1 | 458.00 | 0.0022 |
| *psbB* | *alpina* | 3 | 363.00 | 0.0083 | 0 | 1611.00 | 0 |
|  | *dahurica* | 2 | 363.00 | 0.0055 | 1 | 1611.00 | 0.0009 |
|  | *grandis* | 1 | 363.17 | 0.0028 | 1 | 1611.00 | 0.0009 |
|  | *hirsutissima* | 2 | 363.00 | 0.0055 | 0 | 1611.00 | 0 |
|  | *ludoviciana* | 2 | 363.00 | 0.0055 | 0 | 1611.00 | 0 |
|  | *multifida* | 2 | 363.00 | 0.0055 | 0 | 1611.00 | 0 |
|  | *occidentalis* | 2 | 363.17 | 0.0055 | 1 | 1611.00 | 0.0009 |
| *psbT* | *alpina* | 0 | 25.83 | 0 | 0 | 79.17 | 0 |
|  | *dahurica* | 0 | 25.83 | 0 | 0 | 79.17 | 0 |
|  | *grandis* | 0 | 25.83 | 0 | 0 | 79.17 | 0 |
|  | *hirsutissima* | 0 | 25.83 | 0 | 0 | 79.17 | 0 |
|  | *ludoviciana* | 0 | 25.83 | 0 | 0 | 79.17 | 0 |
|  | *multifida* | 0 | 25.83 | 0 | 0 | 79.17 | 0 |
|  | *occidentalis* | 0 | 25.83 | 0 | 0 | 79.17 | 0 |
| *psbN* | *alpina* | 0 | 31.17 | 0 | 0 | 97.83 | 0 |
|  | *dahurica* | 0 | 31.17 | 0 | 0 | 97.83 | 0 |
|  | *grandis* | 0 | 31.17 | 0 | 0 | 97.83 | 0 |
|  | *hirsutissima* | 0 | 31.17 | 0 | 0 | 97.83 | 0 |
|  | *ludoviciana* | 0 | 31.17 | 0 | 0 | 97.83 | 0 |
|  | *multifida* | 0 | 31.17 | 0 | 0 | 97.83 | 0 |
|  | *occidentalis* | 0 | 31.17 | 0 | 0 | 97.83 | 0 |
| *psbH* | *alpina* | 0 | 55.00 | 0 | 3 | 164.00 | 0.0185 |
|  | *dahurica* | 0 | 54.83 | 0 | 0 | 164.17 | 0 |
|  | *grandis* | 0 | 54.83 | 0 | 0 | 164.17 | 0 |
|  | *hirsutissima* | 0 | 55.00 | 0 | 1 | 164.00 | 0.0061 |
|  | *ludoviciana* | 0 | 55.00 | 0 | 1 | 164.00 | 0.0061 |
|  | *multifida* | 0 | 55.00 | 0 | 1 | 164.00 | 0.0061 |
|  | *occidentalis* | 1 | 55.00 | 0.0184 | 3 | 164.00 | 0.0185 |
| *petB* | *alpina* | 3 | 157.83 | 0.0193 | 2 | 487.17 | 0.0041 |
|  | *dahurica* | 0 | 172.00 | 0 | 0 | 533.00 | 0 |
|  | *grandis* | 1 | 157.83 | 0.0064 | 2 | 487.17 | 0.0041 |
|  | *hirsutissima* | 1 | 157.83 | 0.0064 | 2 | 487.17 | 0.0041 |
|  | *ludoviciana* | 1 | 157.83 | 0.0064 | 2 | 487.17 | 0.0041 |
|  | *multifida* | 1 | 157.83 | 0.0064 | 2 | 487.17 | 0.0041 |
|  | *occidentalis* | 3 | 157.83 | 0.0193 | 2 | 487.17 | 0.0041 |
| *petD* | *alpina* | 5 | 122.33 | 0.0420 | 0 | 378.67 | 0 |
|  | *dahurica* | 0 | 122.33 | 0 | 0 | 378.67 | 0 |
|  | *grandis* | 1 | 122.33 | 0.0082 | 0 | 378.67 | 0 |
|  | *hirsutissima* | 1 | 122.33 | 0.0082 | 0 | 378.67 | 0 |
|  | *ludoviciana* | 1 | 122.33 | 0.0082 | 0 | 378.67 | 0 |
|  | *multifida* | 1 | 122.33 | 0.0082 | 0 | 378.67 | 0 |
|  | *occidentalis* | 3 | 122.33 | 0.0249 | 0 | 378.67 | 0 |
| *rpoA* | *alpina* | 1 | 221.50 | 0.0045 | 3 | 795.50 | 0.0038 |
|  | *dahurica* | 1 | 221.33 | 0.0045 | 1 | 795.67 | 0.0013 |
|  | *grandis* | 1 | 221.33 | 0.0045 | 1 | 795.67 | 0.0013 |
|  | *hirsutissima* | 2 | 221.33 | 0.0090 | 1 | 795.67 | 0.0013 |
|  | *ludoviciana* | 2 | 221.00 | 0.0091 | 2 | 796.00 | 0.0025 |
|  | *multifida* | 2 | 221.33 | 0.0090 | 1 | 795.67 | 0.0013 |
|  | *occidentalis* | 1 | 221.00 | 0.0045 | 5 | 796.00 | 0.0063 |
| *rps11* | *alpina* | 2 | 106.50 | 0.0190 | 0 | 307.50 | 0 |
|  | *dahurica* | 2 | 106.50 | 0.0190 | 0 | 307.50 | 0 |
|  | *grandis* | 2 | 106.17 | 0.0191 | 0 | 307.83 | 0 |
|  | *hirsutissima* | 1 | 106.00 | 0.0095 | 2 | 308.00 | 0.0065 |
|  | *ludoviciana* | 1 | 106.33 | 0.0095 | 1 | 307.67 | 0.0033 |
|  | *multifida* | 2 | 106.33 | 0.0190 | 1 | 307.67 | 0.0033 |
|  | *occidentalis* | 3 | 106.50 | 0.0287 | 1 | 307.50 | 0.0033 |
| *rpl36* | *alpina* | 2 | 25.67 | 0.0823 | 1 | 85.33 | 0.0118 |
|  | *dahurica* | 3 | 26.00 | 0.1253 | 0 | 85.00 | 0 |
|  | *grandis* | 2 | 25.67 | 0.0823 | 0 | 85.33 | 0 |
|  | *hirsutissima* | 2 | 25.67 | 0.0823 | 0 | 85.33 | 0 |
|  | *ludoviciana* | 2 | 25.67 | 0.0823 | 0 | 85.33 | 0 |
|  | *multifida* | 2 | 25.67 | 0.0823 | 0 | 85.33 | 0 |
|  | *occidentalis* | 2 | 25.67 | 0.0823 | 1 | 85.33 | 0.0118 |
| *rps8* | *alpina* | 0 | 101.67 | 0 | 0 | 294.33 | 0 |
|  | *dahurica* | 0 | 101.67 | 0 | 0 | 294.33 | 0 |
|  | *grandis* | 0 | 101.67 | 0 | 0 | 294.33 | 0 |
|  | *hirsutissima* | 0 | 101.67 | 0 | 0 | 294.33 | 0 |
|  | *ludoviciana* | 0 | 101.67 | 0 | 0 | 294.33 | 0 |
|  | *multifida* | 0 | 101.67 | 0 | 0 | 294.33 | 0 |
|  | *occidentalis* | 0 | 101.67 | 0 | 0 | 294.33 | 0 |
| *rpl14* | *alpina* | 0 | 87.17 | 0 | 0 | 278.83 | 0 |
|  | *dahurica* | 0 | 87.17 | 0 | 0 | 278.83 | 0 |
|  | *grandis* | 0 | 87.17 | 0 | 0 | 278.83 | 0 |
|  | *hirsutissima* | 0 | 87.17 | 0 | 0 | 278.83 | 0 |
|  | *ludoviciana* | 0 | 87.17 | 0 | 0 | 278.83 | 0 |
|  | *multifida* | 0 | 87.17 | 0 | 0 | 278.83 | 0 |
|  | *occidentalis* | 0 | 87.17 | 0 | 0 | 278.83 | 0 |
| *rpl16* | *alpina* | 1 | 105.50 | 0.0095 | 0 | 299.50 | 0 |
|  | *dahurica* | 1 | 105.50 | 0.0095 | 0 | 299.50 | 0 |
|  | *grandis* | 1 | 105.50 | 0.0095 | 0 | 299.50 | 0 |
|  | *hirsutissima* | 1 | 105.50 | 0.0095 | 0 | 299.50 | 0 |
|  | *ludoviciana* | 1 | 105.50 | 0.0095 | 0 | 299.50 | 0 |
|  | *multifida* | 1 | 105.50 | 0.0095 | 0 | 299.50 | 0 |
|  | *occidentalis* | 1 | 105.50 | 0.0095 | 0 | 299.50 | 0 |
| *rps3* | *alpina* | 0 | 144.83 | 0 | 0 | 509.17 | 0 |
|  | *dahurica* | 0 | 144.83 | 0 | 0 | 509.17 | 0 |
|  | *grandis* | 0 | 144.83 | 0 | 0 | 509.17 | 0 |
|  | *hirsutissima* | 0 | 144.83 | 0 | 0 | 509.17 | 0 |
|  | *ludoviciana* | 0 | 144.83 | 0 | 0 | 509.17 | 0 |
|  | *multifida* | 0 | 144.83 | 0 | 0 | 509.17 | 0 |
|  | *occidentalis* | 0 | 144.83 | 0 | 0 | 509.17 | 0 |
| *rpl22* | *alpina* | 0 | 106.58 | 0 | 2 | 370.42 | 0.0054 |
|  | *dahurica* | 0 | 117.83 | 0 | 0 | 425.17 | 0 |
|  | *grandis* | 0 | 117.83 | 0 | 0 | 425.17 | 0 |
|  | *hirsutissima* | 0 | 117.08 | 0 | 2 | 419.92 | 0.0048 |
|  | *ludoviciana* | 0 | 117.08 | 0 | 2 | 419.92 | 0.0048 |
|  | *multifida* | 0 | 103.92 | 0 | 2 | 361.08 | 0.0056 |
|  | *occidentalis* | 0 | 117.08 | 0 | 3 | 419.92 | 0.0072 |
| *rps19* | *alpina* | 0 | 55.17 | 0 | 0 | 181.83 | 0 |
|  | *dahurica* | 0 | 55.17 | 0 | 0 | 181.83 | 0 |
|  | *grandis* | 0 | 55.17 | 0 | 0 | 181.83 | 0 |
|  | *hirsutissima* | 0 | 55.17 | 0 | 0 | 181.83 | 0 |
|  | *ludoviciana* | 0 | 55.17 | 0 | 0 | 181.83 | 0 |
|  | *multifida* | 0 | 55.17 | 0 | 0 | 181.83 | 0 |
|  | *occidentalis* | 0 | 55.17 | 0 | 0 | 181.83 | 0 |
| *rpl2* | *alpina* | 0 | 55.17 | 0 | 0 | 181.83 | 0 |
|  | *dahurica* | 0 | 55.17 | 0 | 0 | 181.83 | 0 |
|  | *grandis* | 0 | 55.17 | 0 | 0 | 181.83 | 0 |
|  | *hirsutissima* | 0 | 55.17 | 0 | 0 | 181.83 | 0 |
|  | *ludoviciana* | 0 | 55.17 | 0 | 0 | 181.83 | 0 |
|  | *multifida* | 0 | 55.17 | 0 | 0 | 181.83 | 0 |
|  | *occidentalis* | 0 | 55.17 | 0 | 0 | 181.83 | 0 |
| *rpl23* | *alpina* | 0 | 65.00 | 0 | 0 | 214.00 | 0 |
|  | *dahurica* | 0 | 65.00 | 0 | 0 | 214.00 | 0 |
|  | *grandis* | 0 | 65.00 | 0 | 0 | 214.00 | 0 |
|  | *hirsutissima* | 0 | 65.00 | 0 | 0 | 214.00 | 0 |
|  | *ludoviciana* | 0 | 65.00 | 0 | 0 | 214.00 | 0 |
|  | *multifida* | 0 | 65.00 | 0 | 0 | 214.00 | 0 |
|  | *occidentalis* | 0 | 65.00 | 0 | 0 | 214.00 | 0 |
| *ycf2* | *alpina* | 4 | 1502.25 | 0.0027 | 9 | 5310.75 | 0.0017 |
|  | *dahurica* | 0 | 1504.00 | 0 | 0 | 5318.00 | 0 |
|  | *grandis* | 1 | 1504.58 | 0.0007 | 2 | 5317.42 | 0.0004 |
|  | *hirsutissima* | 0 | 1503.67 | 0 | 7 | 5315.33 | 0.0013 |
|  | *ludoviciana* | 0 | 1504.67 | 0 | 7 | 5317.33 | 0.0013 |
|  | *multifida* | 0 | 1503.33 | 0 | 6 | 5315.67 | 0.0011 |
|  | *occidentalis* | 3 | 1504.25 | 0.0020 | 7 | 5317.75 | 0.0013 |
| *ndhB* | *alpina* | 0 | 365.67 | 0 | 1 | 1110.33 | 0.0009 |
|  | *dahurica* | 0 | 365.50 | 0 | 0 | 1110.50 | 0 |
|  | *grandis* | 0 | 365.67 | 0 | 1 | 1110.33 | 0.0009 |
|  | *hirsutissima* | 0 | 365.67 | 0 | 1 | 1110.33 | 0.0009 |
|  | *ludoviciana* | 4 | 316.42 | 0.0127 | 16 | 1156.58 | 0.0140 |
|  | *multifida* | 0 | 365.67 | 0 | 1 | 1110.33 | 0.0009 |
|  | *occidentalis* | 0 | 365.67 | 0 | 1 | 1110.33 | 0.0009 |
| *rps7* | *alpina* | 0 | 112.83 | 0 | 0 | 353.17 | 0 |
|  | *dahurica* | 0 | 112.83 | 0 | 0 | 353.17 | 0 |
|  | *grandis* | 0 | 112.83 | 0 | 0 | 353.17 | 0 |
|  | *hirsutissima* | 0 | 112.83 | 0 | 0 | 353.17 | 0 |
|  | *ludoviciana* | 0 | 112.83 | 0 | 0 | 353.17 | 0 |
|  | *multifida* | 0 | 112.83 | 0 | 0 | 353.17 | 0 |
|  | *occidentalis* | 0 | 112.83 | 0 | 0 | 353.17 | 0 |
| *ycf1* | *alpina* | 14 | 1182.83 | 0.0119 | 73 | 4451.17 | 0.0166 |
|  | *dahurica* | 6 | 1184.50 | 0.0051 | 30 | 4464.50 | 0.0067 |
|  | *grandis* | 5 | 1187.25 | 0.0042 | 30 | 4470.75 | 0.0067 |
|  | *hirsutissima* | 8 | 1183.83 | 0.0068 | 55 | 4456.17 | 0.0124 |
|  | *ludoviciana* | 9.5 | 1179.50 | 0.0081 | 43.50 | 4433.50 | 0.0099 |
|  | *multifida* | 8.5 | 1183.00 | 0.0072 | 44.5 | 4445.00 | 0.0101 |
|  | *occidentalis* | 10.5 | 1179.00 | 0.0090 | 63.50 | 4428.00 | 0.0145 |
| *rps15* | *alpina* | 1 | 61.00 | 0.0166 | 0 | 209.00 | 0 |
|  | *dahurica* | 0 | 61.00 | 0 | 1 | 209.00 | 0.0048 |
|  | *grandis* | 0 | 60.67 | 0 | 1 | 209.33 | 0.0048 |
|  | *hirsutissima* | 0 | 61.17 | 0 | 1 | 208.83 | 0.0048 |
|  | *ludoviciana* | 0 | 61.00 | 0 | 0 | 209.00 | 0 |
|  | *multifida* | 0 | 61.00 | 0 | 0 | 209.00 | 0 |
|  | *occidentalis* | 1 | 61.00 | 0.0166 | 0 | 209.00 | 0 |
| *ndhH* | *alpina* | 0 | 269.83 | 0 | 0 | 909.17 | 0 |
|  | *dahurica* | 2 | 269.50 | 0.0075 | 0 | 909.50 | 0 |
|  | *grandis* | 0 | 269.83 | 0 | 0 | 909.17 | 0 |
|  | *hirsutissima* | 1 | 269.83 | 0.0037 | 0 | 909.17 | 0 |
|  | *ludoviciana* | 1 | 269.83 | 0.0037 | 0 | 909.17 | 0 |
|  | *multifida* | 1 | 233.83 | 0.0043 | 0 | 795.17 | 0 |
|  | *occidentalis* | 1 | 269.00 | 0.0037 | 2 | 910.00 | 0.0022 |
| *ndhA* | *alpina* | 3 | 271.67 | 0.0111 | 0 | 817.33 | 0 |
|  | *dahurica* | 2 | 271.83 | 0.0074 | 1 | 817.17 | 0.0012 |
|  | *grandis* | 1 | 272.00 | 0.0037 | 0 | 817.00 | 0 |
|  | *hirsutissima* | 1 | 272.00 | 0.0037 | 0 | 817.00 | 0 |
|  | *ludoviciana* | 1 | 272.00 | 0.0037 | 0 | 817.00 | 0 |
|  | *multifida* | 1 | 272.00 | 0.0037 | 0 | 817.00 | 0 |
|  | *occidentalis* | 1 | 272.00 | 0.0037 | 1 | 817.00 | 0.0012 |
| *ndhI* | *alpina* | 1 | 119.17 | 0.0084 | 1 | 402.83 | 0.0025 |
|  | *dahurica* | 1 | 120.75 | 0.0083 | 1 | 407.25 | 0.0025 |
|  | *grandis* | 2 | 121.00 | 0.0167 | 2 | 410.00 | 0.0049 |
|  | *hirsutissima* | 0 | 120.83 | 0 | 3 | 407.17 | 0.0073 |
|  | *ludoviciana* | 0.5 | 120.83 | 0.0041 | 5.5 | 407.17 | 0.0136 |
|  | *multifida* | 1 | 120.50 | 0.0083 | 0 | 410.50 | 0 |
|  | *occidentalis* | 0 | 121.00 | 0 | 2 | 410.00 | 0.0049 |
| *ndhG* | *alpina* | 2 | 128.17 | 0.0158 | 1 | 402.83 | 0.0025 |
|  | *dahurica* | 2 | 127.67 | 0.0158 | 2 | 403.33 | 0.0050 |
|  | *grandis* | 1 | 128.00 | 0.0079 | 1 | 403.00 | 0.0025 |
|  | *hirsutissima* | 2 | 128.33 | 0.0157 | 0 | 402.67 | 0 |
|  | *ludoviciana* | 0 | 41.33 | 0 | 0 | 123.67 | 0 |
|  | *multifida* | 1 | 128.00 | 0.0079 | 1 | 403.00 | 0.0025 |
|  | *occidentalis* | 1 | 128.00 | 0.0079 | 1 | 403.00 | 0.0025 |
| *ndhE* | *alpina* | 2 | 71.00 | 0.0287 | 0 | 232.00 | 0 |
|  | *dahurica* | 0 | 70.67 | 0 | 1 | 232.33 | 0.0043 |
|  | *grandis* | 0 | 71.00 | 0 | 0 | 232.00 | 0 |
|  | *hirsutissima* | 0 | 71.00 | 0 | 0 | 232.00 | 0 |
|  | *ludoviciana* | 0 | 71.00 | 0 | 0 | 232.00 | 0 |
|  | *multifida* | 0 | 71.00 | 0 | 0 | 232.00 | 0 |
|  | *occidentalis* | 1 | 71.00 | 0.0142 | 0 | 232.00 | 0 |
| *psaC* | *alpina* | 1 | 56.50 | 0.0179 | 0 | 186.50 | 0 |
|  | *dahurica* | 1 | 56.50 | 0.0179 | 0 | 186.50 | 0 |
|  | *grandis* | 1 | 56.50 | 0.0179 | 0 | 186.50 | 0 |
|  | *hirsutissima* | 1 | 56.50 | 0.0179 | 0 | 186.50 | 0 |
|  | *ludoviciana* | 1 | 56.50 | 0.0179 | 0 | 186.50 | 0 |
|  | *multifida* | 0 | 56.50 | 0 | 0 | 186.50 | 0 |
|  | *occidentalis* | 1 | 56.83 | 0.0178 | 1 | 186.17 | 0.0054 |
| *ndhD* | *alpina* | 4 | 344.75 | 0.0117 | 8 | 1119.25 | 0.0072 |
|  | *dahurica* | 3 | 344.50 | 0.0088 | 3 | 1119.50 | 0.0027 |
|  | *grandis* | 2 | 344.50 | 0.0058 | 3 | 1119.50 | 0.0027 |
|  | *hirsutissima* | 3 | 344.42 | 0.0088 | 5 | 1119.58 | 0.0045 |
|  | *ludoviciana* | 3 | 344.25 | 0.0088 | 5 | 1119.75 | 0.0045 |
|  | *multifida* | 2 | 344.42 | 0.0058 | 4 | 1119.58 | 0.0036 |
|  | *occidentalis* | 5 | 344.75 | 0.0146 | 10 | 1119.25 | 0.0090 |
| *ccsA* | *alpina* | 4 | 212.83 | 0.0190 | 5 | 744.17 | 0.0067 |
|  | *dahurica* | 2 | 213.08 | 0.0094 | 2 | 749.92 | 0.0027 |
|  | *grandis* | 3 | 213.42 | 0.0142 | 4 | 749.58 | 0.0054 |
|  | *hirsutissima* | 4 | 211.67 | 0.0191 | 2 | 742.33 | 0.0027 |
|  | *ludoviciana* | 4 | 212.17 | 0.0191 | 3 | 744.83 | 0,0040 |
|  | *multifida* | 2 | 163.67 | 0.0123 | 0 | 553.33 | 0 |
|  | *occidentalis* | 4 | 212.17 | 0.0191 | 5 | 744.83 | 0.0067 |
| *ndhF* | *alpina* | 11 | 512.75 | 0.0218 | 5 | 1683.25 | 0.0030 |
|  | *dahurica* | 3 | 511.67 | 0.0059 | 2 | 1681.33 | 0.0012 |
|  | *grandis* | 17.5 | 515.83 | 0.0347 | 33.50 | 1689.17 | 0.0201 |
|  | *hirsutissima* | 4 | 514.67 | 0.0078 | 7 | 1696.33 | 0.0041 |
|  | *ludoviciana* | 4 | 514.83 | 0.0078 | 5 | 1696.17 | 0.0030 |
|  | *multifida* | 4 | 514.83 | 0.0078 | 6 | 1696.17 | 0.0035 |
|  | *occidentalis* | 13 | 509.92 | 0.259 | 8 | 1677.08 | 0.0048 |

synDif: Synonymous sites. Number of mutations

SynPos: Synonymous sites. Number of sites

Ks: Synonymous

Ka: Non-Synonymous

NsynDif: Nonsynonymous sites. Number of mutations

NSynPos: Nonsynonymous sites. Number of sites
