# Supplementary material for: Chloroplast genomes elucidate diversity, phylogeny, and taxonomy of Pulsatilla (Ranunculaceae)
Source: Sci Rep. 2020 Nov 13;10:19781. doi: 10.1038/s41598-020-76699-7 (PMC7666119; doi:10.1038/s41598-020-76699-7)
Supplement: Supplementary file 3 — Supplementary Caption. [file 41598_2020_76699_MOESM3_ESM.doc]

**Figure S1.** Gene rearrangement analyses among *Pulsatilla* species by Mauve alignment.

**Table S1.** Rate of synonymous and non-synonymous substitutions
